# Classification and prediction of protein–protein interaction interface using machine learning algorithm

**DOI:** 10.1038/s41598-020-80900-2

**Published:** 2021-01-19

**Authors:** Subhrangshu Das, Saikat Chakrabarti

**Affiliations:** Structural Biology and Bioinformatics Division, CSIR-Indian Institute of Chemical Biology, Kolkata, WB India

**Keywords:** Computational biology and bioinformatics, Structural biology

## Abstract

Structural insight of the protein–protein interaction (PPI) interface can provide knowledge about the kinetics, thermodynamics and molecular functions of the complex while elucidating its role in diseases and further enabling it as a potential therapeutic target. However, owing to experimental lag in solving protein–protein complex structures, three-dimensional (3D) knowledge of the PPI interfaces can be gained via computational approaches like molecular docking and post-docking analyses. Despite development of numerous docking tools and techniques, success in identification of native like interfaces based on docking score functions is limited. Hence, we employed an in-depth investigation of the structural features of the interface that might successfully delineate native complexes from non-native ones. We identify interface properties, which show statistically significant difference between native and non-native interfaces belonging to homo and hetero, protein–protein complexes. Utilizing these properties, a support vector machine (SVM) based classification scheme has been implemented to differentiate native and non-native like complexes generated using docking decoys. Benchmarking and comparative analyses suggest very good performance of our SVM classifiers. Further, protein interactions, which are proven via experimental findings but not resolved structurally, were subjected to this approach where 3D-models of the complexes were generated and most likely interfaces were predicted. A web server called **P**rotein **C**omplex **P**rediction by **I**nterface **P**roperties (PCPIP) is developed to predict whether interface of a given protein–protein dimer complex resembles known protein interfaces. The server is freely available at http://www.hpppi.iicb.res.in/pcpip/.

## Introduction

Knowledge about protein–protein interactions (PPI) is critical to understand the molecular mechanisms of biochemical processes and cellular pathways. Advent of high-throughput techniques has enabled genome-wide identification of PPIs for quite a few model organisms^1–12^. These large number of experimentally verified as well computationally predicted interactions are collected and systematically stored in various PPI databases, such as molecular interaction database (MINT)^13^, the Human Protein Reference Database (HPRD)^14^, STRING^15^, database of interacting proteins (DIP)^16^, the protein interaction database (IntAct)^17^, etc. These databases contain important information for thousands of interactions, which are regularly used in network based ‘omics’ data analysis. However, most of these interactions lack detailed structural information and thereby making them therapeutically non-viable targets. Under this scenario, computational approaches capable of generating reliable model of protein complexes using protein–protein docking tools can play an important role in complementing the experimental initiatives. However, as these complexes are generated using predictive approaches, objective tests and evaluation tools are required to determine their reliability.

PPI interfaces have been studied extensively to analyze and understand the critical characteristics features that provide affinity, stability and specificity of the complexes. Properties like accessible surface area (ASA) and buried surface area (BSA), interface residue conservation, hydrogen bonds, electrostatic and hydrophobic interactions play major roles in determining the nature of the protein interfaces. Knowledge of interface characteristics has been studied and was further used in identification of protein interfaces or to predict binding specificity^18–38^. Similarly, numerous scoring functions and schemas were developed for improved prediction of protein–protein interfaces out of which only a few could be mentioned due to space restraints^39–74^. Despite all these works, still gap is prevailed between the optimally scoring solutions and the biologically active complexes^75–78^.

Here, we attempt to utilize protein–protein interface properties to establish discernible differences between native-like protein complexes from the non-native ones. We fed the interface properties to a support vector machine (SVM) based classification scheme and trained models to successfully differentiate between native and non-native like complexes derived by protein–protein docking. Machine learning based techniques have been used previously to analyze and predict protein–protein interactions^78–86^. Our exhaustive testing and benchmarking suggest very good performance of our SVM models in distinguishing native and non-native like interfaces for homo and hetero complexes. We also implemented this approach in validating protein interactions, which are proven via experimental findings but the three-dimensional (3D) structure of the complexes and the subsequent interface(s) are yet to be discovered. Finally, we provide a web server platform namely PCPIP to predict whether the interacting interface of a given protein–protein dimer complex significantly resembles known protein interfaces.

## Materials and methods

### Collection and generation of non-redundant protein–protein complex dataset

989 protein–protein dimer complex structures have been derived from protein data bank (PDB)^87^, which were categorized into homo (560) and hetero (429) dimers. Exhaustive redundancy check and filter was applied to these datasets using the CD-HIT^88^ and BLASTp^89^ programs so that no protein complex (both chains) is more than 40% identical to any other complex within the homo or hetero categories, respectively (Table S1, Figure S1). 371 and 346 homo and hetero complexes were obtained after the redundancy check, which were further filtered by successful docking and interface generation criteria (see later for docking and interface filters).

To validate our machine learning based classification system, we have built a separate validation dataset to perform the benchmarking where both the dimer complex and individual monomer structures are available separately (Apo-Holo validation set). This dataset of complex (holo) and non-complex (apo) forms of proteins were collected from a recent report from Viswanathan et al.^90^. Initially, this dataset contained 95 protein–protein hetero complexes (holo-complexes) and their respective monomer structures. However, these 95 complexes were screened to identify native and non-native like interfaces via fraction of conserved native contacts (FNAT) based definition (please see later for details) and we could retrieve docking decoys that passed the FNAT filtration criteria for 32 such complexes (Table S1). This dataset contained 32 protein–protein hetero complexes (holo-complexes) and their respective monomer structures that are separately available as apo proteins (Table S1). Similarly, the 95 complexes were also screened to identify native and non-native interfaces via interface root mean square deviation (iRMSD) based definition (please see later for details) where 68 such complexes were retrieved that passed the iRMSD filtration criteria.

We have also created a negative dataset of 130 protein–protein complexes and subsequent interfaces for proteins that are not supposed to interact according to the Negatome database^91,92^ (Negatome validation set). Further details about the dataset collection are provided in supplementary information file.

Protein and/or domain structures for which experimentally validated protein–protein interaction is reported in the STRING database 10.5^15^ were also collected individually from the PDB database. Protein–protein interaction pairs for which individual monomer structures are available were selected randomly and were further utilized to generate probable dimer structures using protein–protein docking via the PatchDock program^93^. 32 such docked model complexes (STRING dataset; ten for each complex; total: 320) were generated and further evaluated by our machine learning based protein–protein interface prediction algorithm.

### Generation of native and non-native like protein–protein complexes and their interfaces

Known protein dimer complexes were utilized to create native and non-native like interfaces. Constituent monomers of the dimer complexes were separated and docked using the PatchDock protein–protein docking software and the resultant docked solutions were screened to create native and non-native like complexes based on the following criteria. Fraction of conserved native contacts (FNAT) usually provides a fraction of the common residues at interface of docked interface with respect to that from the original complex whereas interface root mean square deviation (iRMSD) compares the actual orientation of the interface forming residues between the docked and original complexes. Hence, FNAT and iRMSD complement each other in their approach of evaluation of a predicted interface.

#### FNAT based categorization

Fraction of conserved native contacts (FNAT) is the number of native (correct) residue–residue contacts in the docked complex divided by the number of contacts in the original complex. So, FNAT reflects the overlap between the original and docked complex interfaces while a FNAT value 1.0 indicates complete overlap between the two. In our training and testing models, the original complex and the docked complexes with FNAT > 0.8 were regarded as true or native like complexes for each dimer whereas false/non-native like complexes were identified using four separate FNAT thresholds, (a) FNAT ≤ 0.25 (highly distinguishable from the native like complexes), (b) FNAT > 0.25 and ≤ 0.5, (moderately distinguishable from the native like complexes) and (c) FNAT > 0.5 and ≤ 0.8 (weakly distinguishable from the native like complexes), and (d) FNAT ≤ 0.8 (mixed), respectively.

Similar to the training–testing dataset, we have used the same four different FNAT thresholds to define the non-native like complexes for the benchmarking dataset (Apo-Holo validation set) also.

#### iRMSD based categorization

Interface root mean square deviation (iRMSD) is the root mean square deviation between the residues of both chains at the interface region. Similar to the FNAT based protocol, native and non-native sets were also identified based on iRMSD where original complex along with one docked complex with iRMSD < 5 Å with respect to the original complex were regarded as native like complex for each dimer whereas non-native like complexes were identified with four different thresholds, (a) iRMSD ≥ 15 Å (highly distinguishable from the native like complexes), (b) iRMSD ≥ 10 Å and < 15 Å, (moderately distinguishable from the native like complexes) and (c) iRMSD ≥ 5 Å and < 10 Å (weakly distinguishable from the native like complexes), and (d) iRMSD ≥ 5 Å (mixed), respectively.

Native and non-native like complexes (Table S1 and Figure S2) categorized according to the criteria explained above with 1:5 ratio (2 native and 10 non-native samples, respectively) were further used for classification purpose.

### Calculation of PPI interface properties

PISA software^94,95^ was used to calculate structural and chemical properties of the macromolecular interfaces such as accessible/buried surface area, free energy of dissociation, presence/absence of hydrogen bond and salt bridges, etc. Further details are provided in supplementary information file. Please check Table S2 for a full list of features that were used for machine leaning method based classification between native and non-native like protein–protein interaction complexes.

### Classification of the PPI interfaces via SVM

Support vector machine (SVM) is a supervised model used for classification by analyzing given features with associated learning algorithms. Here, we have used a radial basis kernel function (RBF) via 100-fold cross validation method where 100 times randomly selected 80% of the whole data has been used to train the SVM model and the rest 20% has been used as test data. Performance on the test models was measured using average of the hundred random trials. LibSVM^96^ was used to build the classifier models.

For both FNAT and iRMSD based categorizations, 100 fold randomized selection of the training (80% data) and testing (20% data) followed by SVM based classification trials were performed for each of the native and non-native complex threshold criterion. Figure S3 provides an overview of the various training and testing SVM runs employed in this study using multiple categories of native and non-native like protein–protein complexes while Table S3 provides the kernel function and other relevant parameters of the various SVM models.

### Benchmarking and comparison of performance

We have used separate set of complexes in order to check the efficacy of our SVM models in correctly identifying native and non-native complexes. Previously described native and non-native complexes within the Apo-Holo validation set were tested against the 100 SVM models built with 80% training data. Based on the definition of native and non-native like complexes using FNAT and iRMSD categorizations, each set containing highly, moderately, weakly, and mixed distinguishable non-native like complexes from the native like complexes were tested against the corresponding 100 models of the training data sets. Average sensitivity, specificity, precision, and F1 score from the 100 runs was recorded.

The separate datasets of Apo-Holo heterodimers using the mixed distinguishable non-native like complex thresholds [FNAT-native (FNAT > 0.8): 64 and non-native (FNAT ≤ 0.8): 320 and iRMSD-native (iRMSD < 5 Å): 134 and non-native (iRMSD ≥ 5 Å):  680] (Table S1) were used as input to the CCharPPI online server^97^ where 10 different composite scoring functions such as ZRANK, ZRANK2, ROSETTADOCK, PYDOCK, FIREDOCK, PISA score, CP_PIE, and SIPPER were applied on each complex and the corresponding scores were obtained. Individual receiver operating characteristic (ROC) plot was created by calculating the true positive rate (TPR; sensitivity) and false positive rate (FPR; 1-specificity) for each scoring function.

Additionally, 130 protein pairs extracted from the Negatome database^91,92^ were subjected to PatchDock to generate biologically non-feasible protein complexes. 5 docked decoy complexes were collected for each query pairs following the criteria described in supplementary information file. This dataset (Negatome validation set) was used to validate the false prediction rate of the SVM classifier.

In absence of reference complex structure, we selected top ten docking solutions according to the PatchDock docking score for each 31 protein–protein interactions enlisted within the STRING dataset, which was further subjected to interface feature generation followed by SVM prediction using the heterodimer training model.

Standard performance metrics such as sensitivity, specificity, precision, F1 score, Mathew’s correlation coefficient (MCC), etc. were calculated along with the ROC statistics to measure the performance of our models in different scenario. Please see supplementary information file for more details.

### Development of the webserver

We have developed a web based server named, “PCPIP (Protein Complex Prediction by Interface Properties)” where the classification and prediction schemes were embedded within a web module. Given a protein–protein complex, the PCPIP server would be able to predict whether the interacting interface resembles significantly with known protein interfaces. The server is available via http://www.hpppi.iicb.res.in/pcpip/ and is developed on PHP and CGI-PERL platform.

The server has two input options, single and batch mode, respectively. In the single mode option, single protein–protein complex (homo or hetero dimer) file saved in standard PDB format can be uploaded to check whether the interacting interface, if there is any, resembles the interfaces extracted from the native protein–protein complexes or not.

SVM models for both homo and hetero dimer along with their interface features are kept as background search models against which the uploaded protein complex can be testified based on FNAT and iRMSD criteria.

## Results

### Protein–protein interface properties to differentiate between native and non-native like interfaces

**Figure 1:**
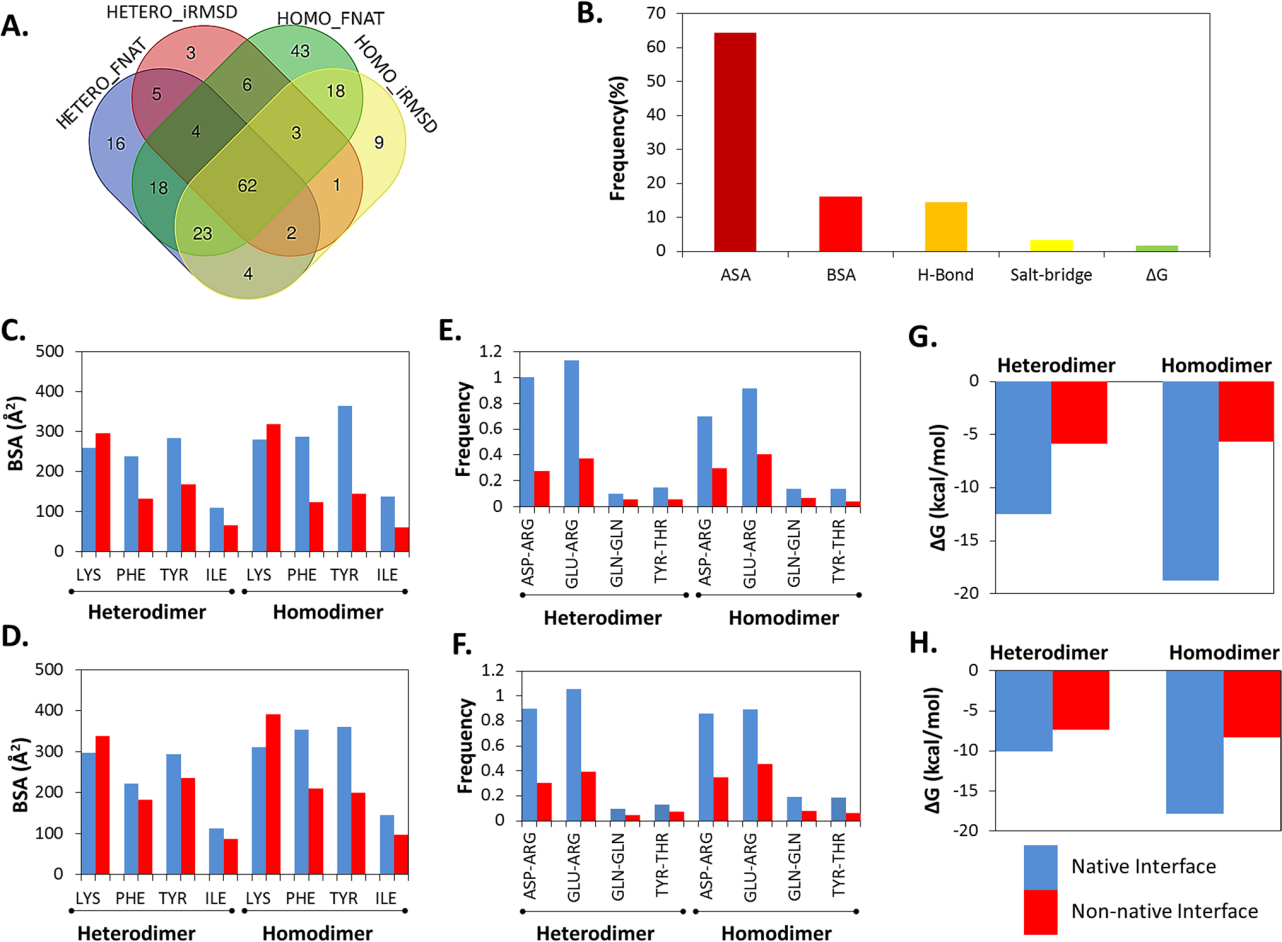
Comparison of protein–protein interaction interface properties. (**A**) The overlap among interface properties that were showing statistically significant (*p ≤ *0.01) differences between the native and non-native like complexes, categorized either by FNAT and iRMSD criteria. HETERO_FNAT and HETERO_iRMSD provide numbers of significantly different interface properties for heterodimers while HOMO_FNAT and HOMO_iRMSD provide numbers of significantly different interface properties for homodimers native and non-native like complexes, respectively. FNAT, fraction of conserved native contacts. iRMSD, interface root mean square deviation. (**B**) The distribution of the common interface properties that showed statistically significant (*p ≤ *0.01) differences between all the native and non-native like complexes. ASA, accessible surface area. BSA, buried surface area. H-bond, hydrogen bonds. (**C**,**D**) plot the buried surface area (BSA) of the two amino acids that possessed significantly different BSA at the native interfaces compared to the non-native ones identified based on FNAT (**C**) and iRMSD (**D**) definitions, respectively. (**E**,**F**) show the hydrogen bond forming amino acid pairs that are found to be significantly higher at the native interfaces compared to the non-native ones identified based on FNAT (**E**) and iRMSD (**F**) based definitions, respectively. (**G**,**H**) plot the average binding energy represented by ΔG for the native and non-native interfaces identified based on FNAT (**G**) and iRMSD (**H**) based definitions, respectively.

PPI interface properties that were showing statistically significant (*p* ≤ 0.01) differences between the native and non-native like complexes, categorized either by FNAT or iRMSD criteria were compared (Fig. 1A). 62 such features were common in homo and heterodimer. A large fraction of the distinguishable features represent accessible surface area (ASA) of amino acids located at the native and non-native like interfaces (Fig. 1B and Figure S4). However, only phenylalanine (PHE), tyrosine (TYR), and isoleucine (ILE) possess significantly higher buried surface area (BSA) in native interfaces whereas lysine (LYS) possesses significantly lower buried surface area (BSA) in native interfaces (Fig. 1C,D). Hydrogen bonds between aspartate-arginine (ASP-ARG) and glutamate-arginine (GLU-ARG) were found to be significantly more in native interfaces (Fig. 1E,F) whereas all the native interfaces were found be significantly more stable with respect to binding energy (Fig. 1G,H). Abundance of negatively charged amino acids (ASP and GLU), serine (SER), threonine (THR) and cysteine (CYS) are relatively lower at the native interfaces of hetero complexes with respect to non-native interfaces whereas positively charged ARG and HIS are relatively higher at the native interfaces of homo complexes, respectively (Figure S5). In addition, frequencies of ALA, GLY, PRO, ASN, GLN, LYS, HIS, PHE, TRP, and TYR are also found to be different between native and non-native interfaces (Figure S5). These observations indicate presence of discernable differences between them and advocates the utilization of the interface features to classify and predict native PPI interfaces.

### Classification of protein–protein interaction interfaces via SVM

Support vector machine (SVM) was used for classification with 100-fold cross validation approach. Table 1 provides mean test accuracies for homodimer and heterodimer complexes, respectively. 100 fold randomized selection of the training (80% data) and testing (20% data) samples followed by SVM based classification trials were performed for each of the native and non-native complex threshold criterion. Classifications were performed with different training models where non-native like interfaces were selected with various FNAT thresholds, (a) FNAT ≤ 0.25 (highly distinguishable from the native like complexes), (b) FNAT ≥ 0.25 and ≤ 0.5, (moderately distinguishable from the native like complexes) and (c) FNAT ≥ 0.5 and ≤ 0.8 (weakly distinguishable from the native like complexes), respectively with respect to the original complex. Similar 100 fold classification was also performed using native and non-native like complexes defined by iRMSD where non-native like complexes were identified with three thresholds, (a) iRMSD ≥ 15 Å (highly distinguishable from the native like complexes), (b) iRMSD ≥ 10 Å and ≤ 15 Å, (moderately distinguishable from the native like complexes) and (c) iRMSD ≥ 5 Å and ≤ 10 Å (weakly distinguishable from the native like complexes), respectively.

**Table 1:** Mean test and train accuracies for prediction of protein–protein interaction interfaces.

PPI category	FNAT							
	NC > 0.80; NNC ≤ 0.25		NC > 0.80; NNC < 0.25 and ≤ 0.5		NC > 0.80; NNC < 0.50 and ≤ 0.80		NC > 0.80; NNC ≤ 0.80	
	Mean test accuracy (%)	Mean test AUC	Mean test accuracy (%)	Mean test AUC	Mean test accuracy (%)	Mean test AUC	Mean test accuracy (%)	Mean test AUC
Homo	99.28 ± 0.41	0.99 ± 0.00	98.78 ± 0.51	0.99 ± 0.00	97.50 ± 0.60	0.99 ± 0.00	98.52 ± 0.50	0.99 ± 0.00
Hetero	99.04 ± 0.49	0.99 ± 0.00	98.31 ± 0.71	0.99 ± 0.00	96.93 ± 0.961	0.99 ± 0.00	98.05 ± 0.55	0.99 ± 0.00

PPI category	iRMSD							
	NC < 5 Å; NNC ≥ 15 Å		NC < 5 Å; NNC ≥ 10 Å and < 15 Å		NC < 5 Å; NNC ≥ 5 Å and < 10 Å		NC < 5 Å; NNC ≥ 5 Å	
	Mean test accuracy (%)	Mean test AUC	Mean test accuracy (%)	Mean test AUC	Mean test accuracy (%)	Mean test AUC	Mean test accuracy (%)	Mean test AUC
Homo	97.55 ± 1.34	0.99 ± 0.00	97.12 ± 1.15	0.99 ± 0.00	94.90647 ± 1.14	0.98 ± 0.00	97.00 ± 0.92	0.99 ± 0.00
Hetero	96.66 ± 0.87	0.99 ± 0.00	96.82 ± 0.59	0.99 ± 0.00	96.83 ± 0.65	0.99 ± 0.00	97.37 ± 0.53	0.99 ± 0.00

NC, native cutoff; NNC, non-native cutoff. Mean and SD were calculated from 100 randomized cross-validations using the 20% testing datasets.

Our SVM models performed quite well and yielded very good performances with all categories of non-native like protein–protein complexes categorized by both FNAT and iRMSD definitions (Table 1). Utilization of both of these criteria adds more reliability to the process of evaluation of PPI interface comparison.

### Benchmarking and comparative validation

As mentioned before, for benchmarking and validation we have used Apo-Holo-validation dataset comprising of 32 (FNAT) and 68 (iRMSD) dimer complexes for which individual monomer structures are also available separately. Monomers were docked using the PatchDock protein–protein docking software^93^ and native and non-native like complexes were generated using various ranges of FNAT and iRMSD based criteria. Original and all docking solutions with FNAT ≥ 0.8 were regarded as native like complexes whereas solutions with FNAT ≤ 0.25, FNAT ≥ 0.25 and ≤ 0.5, FNAT ≥ 0.50 and ≤ 0.80, and FNAT < 0.80, respectively were considered as non-native like complexes. Similarly, Original and all docking solutions with iRMSD < 5 Å were regarded as native like complexes whereas solutions with iRMSD ≥ 15 Å, iRMSD ≥ 10 Å and ≤ 15 Å, iRMSD ≥ 5 Å and ≤ 10 Å, respectively were considered as non-native like complexes. Native and non-native like complexes from this validation datasets were mixed together and the classifier was asked to differentiate the native and non-native ones correctly based on their interface features. Performances of the classifiers were measured using standard parameters. Table 2, shows the performance measure values estimated for a range of probability threshold (0.50–0.95). It is clearly reflected that the performances of the individual models are quite good.

**Table 2:** Benchmarking results for Apo-Holo datasets using the Hetero FNAT and iRMSD models.

Actual native	Actual non-native		Probability threshold	Accuracy	TP	TN	FP	FN	TPR (sensitivity)	TNR (specificity)	NPV	Precision	F1 score	MCC
**Apo-Holo dataset defined by FNAT (native: original and one with FNAT ≥ 0.80 overlap and non-native: ten with FNAT ≤ 0.25 overlap with original complex interface)**														
FNAT_Model (native: original and one from FNAT** ≥ **0.80 overlap and non-native: FNAT** ≤ **0.25 overlap with original complex interface; ratio: 1:5)														
56	280		0.50	93.738	44.340	270.620	9.380	11.660	0.792	0.966	0.959	0.826	0.808	0.771
56	280		0.55	93.640	43.290	271.340	8.660	12.710	0.773	0.969	0.955	0.834	0.802	0.765
56	280		0.60	93.479	42.070	272.020	7.980	13.930	0.751	0.971	0.951	0.841	0.793	0.757
56	280		0.65	93.271	40.690	272.700	7.300	15.310	0.727	0.974	0.947	0.848	0.782	0.746
56	280		0.70	93.024	39.150	273.410	6.590	16.850	0.699	0.976	0.942	0.857	0.769	0.734
56	280		0.75	92.735	37.340	274.250	5.750	18.660	0.667	0.979	0.936	0.867	0.753	0.720
56	280		0.80	92.271	35.040	274.990	5.010	20.960	0.626	0.982	0.929	0.876	0.729	0.699
56	280		0.85	91.688	32.400	275.670	4.330	23.600	0.579	0.985	0.921	0.883	0.698	0.673
56	280		0.90	90.914	29.080	276.390	3.610	26.920	0.519	0.987	0.911	0.890	0.655	0.637
56	280		0.95	89.756	24.350	277.230	2.770	31.650	0.435	0.990	0.898	0.899	0.585	0.581
**Apo-Holo dataset defined by FNAT (native: original and one with FNAT ≥ 0.80 overlap and non-native: ten with FNAT ≥ 0.25 and ≤ 0.50 overlap with original complex interface)**														
FNAT_Model (native: original and one from FNAT** ≥ **0.80 overlap and non-native: FNAT ≥ 0.25 and ≤ 0.5 overlap with original complex interface; ratio: 1:5)														
10	50		0.50	91.467	4.970	49.910	0.090	5.030	0.497	0.998	0.909	0.985	0.659	0.664
10	50		0.55	91.417	4.910	49.940	0.060	5.090	0.491	0.999	0.908	0.990	0.655	0.662
10	50		0.60	91.300	4.810	49.970	0.030	5.190	0.481	0.999	0.906	0.995	0.647	0.657
10	50		0.65	91.233	4.750	49.990	0.010	5.250	0.475	1.000	0.905	0.998	0.642	0.655
10	50		0.70	90.967	4.590	49.990	0.010	5.410	0.459	1.000	0.902	0.998	0.627	0.643
10	50		0.75	90.750	4.450	50.000	0.000	5.550	0.445	1.000	0.900	1.000	0.613	0.633
10	50		0.80	90.383	4.230	50.000	0.000	5.770	0.423	1.000	0.897	1.000	0.592	0.616
10	50		0.85	89.867	3.920	50.000	0.000	6.080	0.392	1.000	0.892	1.000	0.559	0.591
10	50		0.90	88.883	3.330	50.000	0.000	6.670	0.333	1.000	0.883	1.000	0.493	0.542
10	50		0.95	87.133	2.280	50.000	0.000	7.720	0.228	1.000	0.866	1.000	0.365	0.444
**Apo-Holo dataset defined by FNAT (native: original and one with FNAT ≥ 0.80 overlap and non-native: ten with FNAT ≥ 0.50 and ≤ 0.80 overlap with original complex interface)**														
FNAT_Model (native: original and one from FNAT** ≥ **0.80 overlap and non-native: FNAT** ≥ **0.50 and ≤ 0.80 overlap with original complex interface; ratio: 1:5)														
20	100	0.50		78.083	15.33	78.37	21.63	4.67	0.767	0.784	0.944	0.416	0.538	0.444
20	100	0.55		78.592	14.51	79.8	20.2	5.49	0.726	0.798	0.936	0.419	0.530	0.430
20	100	0.60		79.108	13.69	81.24	18.76	6.31	0.685	0.812	0.928	0.423	0.521	0.417
20	100	0.65		79.650	13.07	82.51	17.49	6.93	0.654	0.825	0.923	0.428	0.516	0.409
20	100	0.70		80.108	12.17	83.96	16.04	7.83	0.609	0.840	0.915	0.432	0.503	0.394
20	100	0.75		80.467	10.99	85.57	14.43	9.01	0.550	0.856	0.905	0.431	0.482	0.370
20	100	0.80		80.842	9.94	87.07	12.93	10.06	0.497	0.871	0.897	0.432	0.460	0.349
20	100	0.85		80.692	8.35	88.48	11.52	11.65	0.418	0.885	0.884	0.416	0.415	0.303
20	100	0.90		80.967	6.84	90.32	9.68	13.16	0.342	0.903	0.873	0.409	0.370	0.265
20	100	0.95		81.400	4.87	92.81	7.19	15.13	0.244	0.928	0.860	0.407	0.299	0.213
**Apo-Holo dataset defined by FNAT (native: original and one with FNAT ≥ 0.80 overlap and non-native: ten with FNAT < 0.80 overlap with original complex interface)**														
FNAT_Model (native: original and one from FNAT** ≥ **0.80 overlap and non-native: FNAT < 0.80 overlap with original complex interface; ratio: 1:5)														
64	320	0.50		89.547	46.48	297.38	22.62	17.52	0.726	0.929	0.944	0.674	0.698	0.636
64	320	0.55		89.672	44.82	299.52	20.48	19.18	0.700	0.936	0.940	0.687	0.693	0.631
64	320	0.60		89.797	43.23	301.59	18.41	20.77	0.675	0.942	0.936	0.703	0.688	0.627
64	320	0.65		89.755	41.3	303.36	16.64	22.7	0.645	0.948	0.930	0.714	0.677	0.618
64	320	0.70		89.784	39.47	305.3	14.7	24.53	0.617	0.954	0.926	0.730	0.667	0.611
64	320	0.75		89.695	37.18	307.25	12.75	26.82	0.581	0.960	0.920	0.746	0.652	0.600
64	320	0.80		89.563	34.52	309.4	10.6	29.48	0.539	0.967	0.913	0.767	0.632	0.586
64	320	0.85		89.357	31.8	311.33	8.67	32.2	0.497	0.973	0.906	0.789	0.608	0.570
64	320	0.90		89.013	28.29	313.52	6.48	35.71	0.442	0.980	0.898	0.817	0.572	0.548
64	320	0.95		88.292	23.05	315.99	4.01	40.95	0.360	0.987	0.885	0.855	0.505	0.506
**Apo-Holo dataset defined by iRMSD (native: original and one with iRMSD ≤ 5 Å overlap and non-native: ten with iRMSD > 15 Å with original complex interface)**														
iRMSD_Model (native: original and one from iRMSD** ≤ **5 Å overlap and non-native: iRMSD > 15 Å overlap with original complex interface; ratio: 1:5)														
98	490		0.5	80.2	402.44	87.56	17.8	82.08	0.82	0.82	0.49	0.96	0.61	0.53
98	490		0.55	76.88	421.08	68.92	21.12	84.69	0.78	0.86	0.54	0.95	0.63	0.56
98	490		0.6	73.96	433.36	56.64	24.04	86.28	0.75	0.88	0.57	0.95	0.65	0.58
98	490		0.65	69.76	442.08	47.92	28.24	87.05	0.71	0.90	0.60	0.94	0.65	0.58
98	490		0.7	66.28	447.2	42.8	31.72	87.33	0.68	0.91	0.62	0.93	0.64	0.57
98	490		0.75	61.88	455.56	34.44	36.12	88.00	0.63	0.93	0.65	0.93	0.64	0.57
98	490		0.8	57	464.84	25.16	41	88.75	0.58	0.95	0.70	0.92	0.63	0.57
98	490		0.85	51.12	472.44	17.56	46.88	89.04	0.52	0.96	0.75	0.91	0.61	0.57
98	490		0.9	43.6	479.68	10.32	54.4	88.99	0.44	0.98	0.82	0.90	0.57	0.55
98	490		0.95	31.76	486.84	3.16	66.24	88.20	0.32	0.99	0.91	0.88	0.48	0.50
**Apo-Holo dataset defined by iRMSD (native: original and one with iRMSD ≤ 5 Å overlap and non-native: ten with iRMSD > 10 Å and ≤ 15 Å overlap with original complex interface)**														
iRMSD_Model (native: original and one from iRMSD** ≤ **5 Å overlap and non-native: iRMSD > 5 Å and ≤ 10 Å overlap with original complex interface; ratio: 1:5)														
134	680		0.5	126.52	483.76	196.24	7.48	74.97	0.94	0.71	0.39	0.98	0.55	0.50
134	680		0.55	124.56	501.56	178.44	9.44	76.92	0.93	0.74	0.41	0.98	0.57	0.51
134	680		0.6	122.6	520.8	159.2	11.4	79.04	0.91	0.77	0.44	0.98	0.59	0.53
134	680		0.65	120.16	532.4	147.6	13.84	80.17	0.90	0.78	0.45	0.97	0.60	0.54
134	680		0.7	117.4	551	129	16.6	82.11	0.88	0.81	0.48	0.97	0.62	0.55
134	680		0.75	114.04	565.76	114.24	19.96	83.51	0.85	0.83	0.50	0.97	0.63	0.56
134	680		0.8	109.28	583.24	96.76	24.72	85.08	0.82	0.86	0.53	0.96	0.64	0.57
134	680		0.85	102.6	604.6	75.4	31.4	86.88	0.77	0.89	0.58	0.95	0.66	0.59
134	680		0.9	94.08	628.36	51.64	39.92	88.75	0.70	0.92	0.65	0.94	0.67	0.61
134	680		0.95	75.84	653	27	58.16	89.54	0.57	0.96	0.74	0.92	0.64	0.59
**Apo-Holo dataset defined by iRMSD (native: original and one with iRMSD ≤ 5 Å overlap and non-native: ten with iRMSD > 5 Å and ≤ 10 Å overlap with original complex interface)**														
iRMSD_Model (native: original and one from iRMSD** ≤ **5 Å overlap and non-native: iRMSD > 10 Å and ≤ 15 Å overlap with original complex interface; ratio: 1:5)														
134	680	0.5		129.52	380.88	299.12	4.48	62.70	0.97	0.56	0.30	0.99	0.46	0.39
134	680	0.55		128.04	397.72	282.28	5.96	64.59	0.96	0.58	0.31	0.99	0.47	0.40
134	680	0.6		126.68	418.12	261.88	7.32	66.93	0.95	0.61	0.33	0.98	0.49	0.42
134	680	0.65		124.2	436.12	243.88	9.8	68.84	0.93	0.64	0.34	0.98	0.50	0.43
134	680	0.7		122.24	457.04	222.96	11.76	71.16	0.91	0.67	0.36	0.97	0.51	0.44
134	680	0.75		118.08	479.52	200.48	15.92	73.42	0.88	0.71	0.37	0.97	0.52	0.45
134	680	0.8		113	503.64	176.36	21	75.75	0.84	0.74	0.39	0.96	0.54	0.45
134	680	0.85		105.48	532.28	147.72	28.52	78.35	0.79	0.78	0.42	0.95	0.55	0.46
134	680	0.9		95.28	568.56	111.44	38.72	81.55	0.71	0.84	0.47	0.94	0.56	0.47
134	680	0.95		75.92	615.28	64.72	58.08	84.91	0.57	0.90	0.55	0.91	0.55	0.47
**Apo-Holo dataset defined by iRMSD (native: original and all with iRMSD ≤ 5 Å overlap and non-native: ten with iRMSD > 5 Å overlap with original complex interface)**														
iRMSD_Model (native: original and one from iRMSD** ≤ **5 Å overlap and non-native: iRMSD > 5 Å overlap with original complex interface; ratio: 1:5)														
134	680	0.5		124.44	566.56	113.44	9.56	84.89	0.93	0.83	0.53	0.98	0.67	0.62
134	680	0.55		122.68	578.56	101.44	11.32	86.15	0.92	0.85	0.55	0.98	0.69	0.64
134	680	0.6		120.12	587.6	92.4	13.88	86.94	0.90	0.86	0.57	0.98	0.70	0.64
134	680	0.65		117.28	595.6	84.4	16.72	87.58	0.88	0.88	0.59	0.97	0.70	0.65
134	680	0.7		114.32	606.12	73.88	19.68	88.51	0.85	0.89	0.61	0.97	0.71	0.66
134	680	0.75		110.6	617.96	62.04	23.4	89.50	0.83	0.91	0.64	0.96	0.72	0.67
134	680	0.8		104.72	628.36	51.64	29.28	90.06	0.78	0.92	0.67	0.96	0.72	0.67
134	680	0.85		97.88	637.04	42.96	36.12	90.29	0.73	0.94	0.70	0.95	0.71	0.66
134	680	0.9		87.84	648.8	31.2	46.16	90.50	0.66	0.95	0.74	0.93	0.70	0.64
134	680	0.95		69.96	664.28	15.72	64.04	90.20	0.52	0.98	0.82	0.91	0.64	0.60

**Figure 2:**
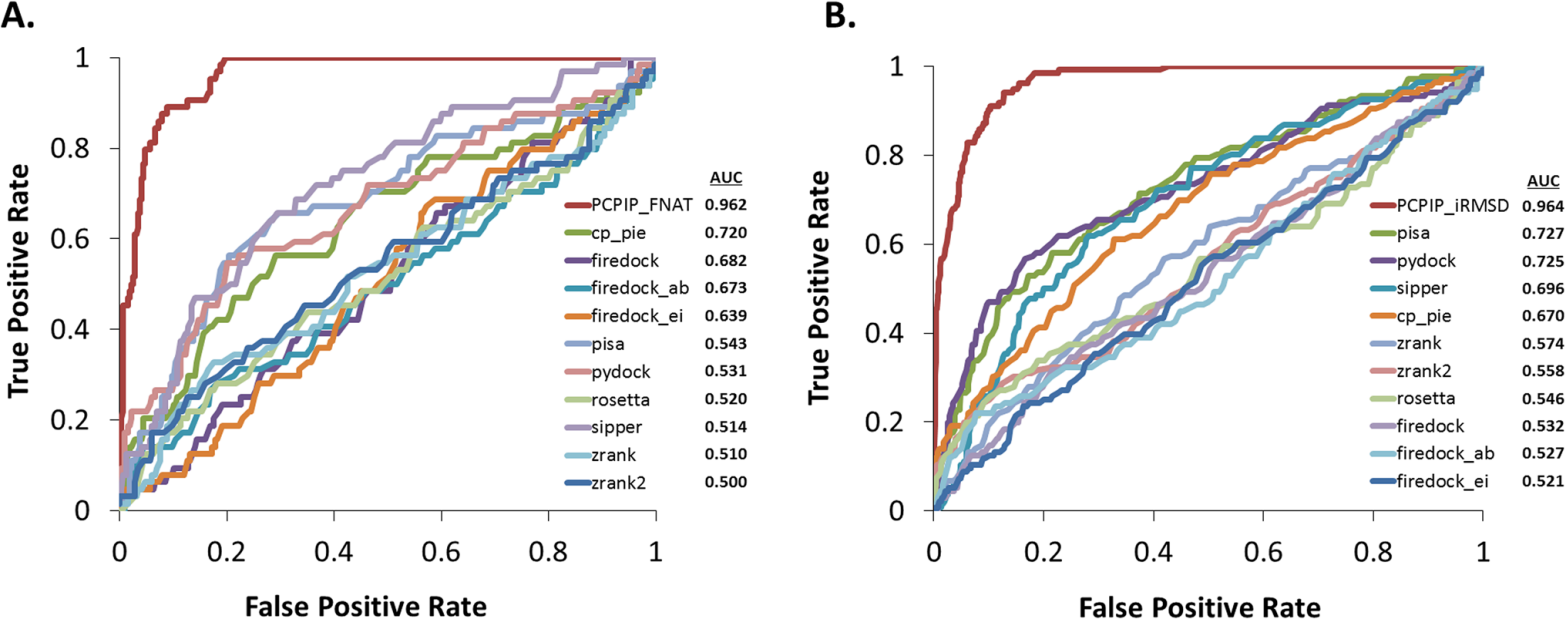
Comparison of prediction performances. The prediction performances of the SVM based prediction models (PCPIP_FNAT and PCPIP_iRMSD) for native and non-native like complexes from the Apo-Holo dataset were compared against 10 different types of scoring functions. Receiver operating characteristic (ROC) plots were created by calculating the true positive rate (TPR; Y axes) and false positive rate (FPR; X axes). PCPIP stands for Protein Complex Prediction by Interface Properties. Area under curve (AUC) values for each of the methods is also provided. Benchmarking was performed using the FNAT (**A**) and iRMSD (**B**) definitions based sub-datasets from the Apo-Holo validation set where native-like complexes were defined by FNAT > 0.8 and iRMSD < 5 Å, respectively and non-native like complexes were identified using FNAT ≤ 0.8 and iRMSD ≥ 15 Å,  respectively.

The datasets showed in Table 2 were used in CCharPPI online server^97^ where 10 different composite scoring functions were applied on each complex and the corresponding scores are obtained. ROC plots were created (Fig. 2) by calculating the true positive rate (TPR) and false positive rate (FPR; 1-specificity). Figure 2 clearly demonstrates much better performance of our FNAT (PCPIP_FNAT) and iRMSD (PCPIP_iRMSD) based prediction models in predicting complexes correctly. Hence, in this apparently difficult datasets our method performed much better compared to the other methods.

**Figure 3:**
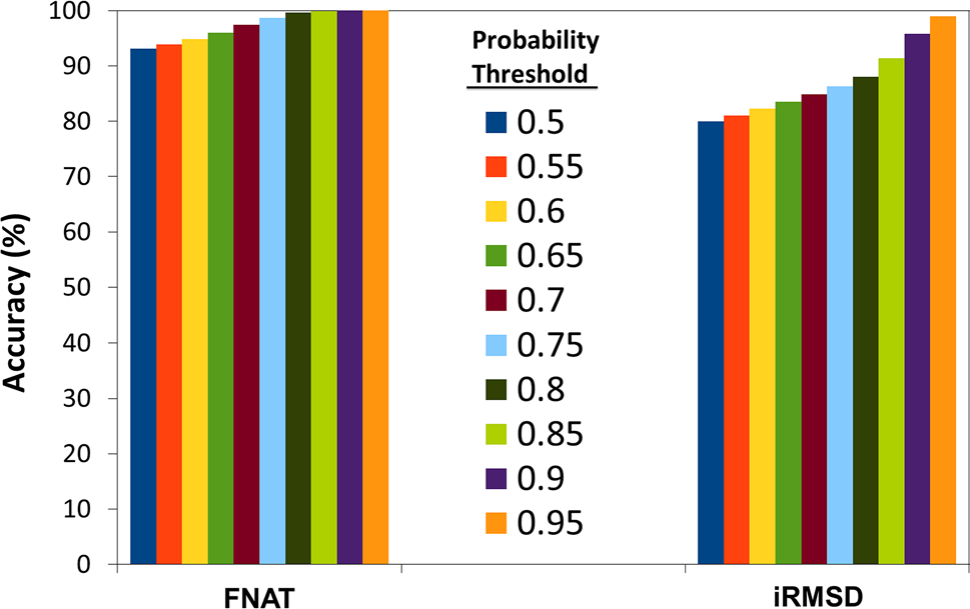
Verification of prediction accuracy. Percentage of correctly predicted non-native hetero complexes extracted from the Negatome dataset using both FNAT and iRMSD definitions are plotted. Accuracies are plotted as bar diagram for each probability threshold cutoff marked by different colors.

Further, the accuracy of all 100 randomly generated training models was testified using a test dataset of complexes that are not supposed to be formed physiologically. Analyzing top 25 cases, it is evident that at all probability thresholds (0.5–0.95) more than 90% of the Negatome complexes were predicted as false by FNAT model where iRMSD model is taking 0.85 probability threshold to reach 90% accuracy (Fig. 3).

### Applications of the prediction algorithm

**Figure 4:**
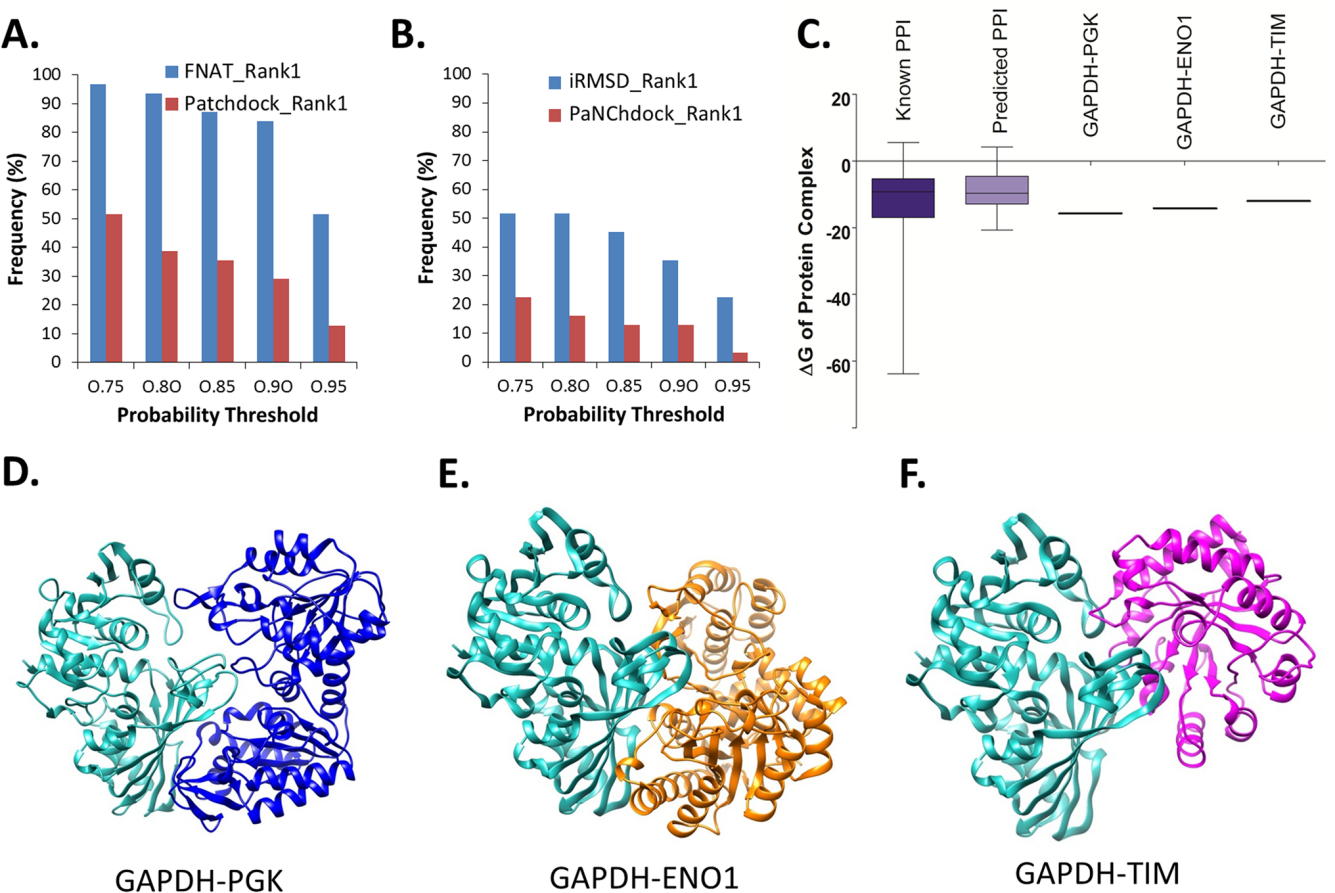
Prediction of probable interaction surface. (**A**,**B**) The frequency of the probability threshold scores within the FNAT (**A**) and iRMSD (**B**) based top ranked solutions in comparison with same derived from PatchDock based top ranked solutions. (**C**) Box plot representation of the binding energy of the protein–protein interaction interface (represented via ΔG) of the 12 docked complexes that were commonly predicted by both FNAT and iRMSD models with highest reliability (probability threshold ≥ 0.95) along with the same obtained from the known 3D structures of the heterodimer complexes. ΔGs of the three representative complexes of GAPDH-PGK, GAPDH-ENO1, and GAPDH-TIM are also plotted. (**D**,**F**) show the 3D cartoon representations of the complexes where GAPDH is shown in cyan and the PGK1, ENO1, and TIM are shown in purple, orange, and blue, respectively.

We collected 32 STRING suggested, experimentally verified protein–protein interactions and the complexes were modeled using the PatchDock based protein docking algorithm where monomer structures/domains were collected from the PDB^87^. Docking solution with highest probability threshold score was considered as the top ranked prediction based on FNAT and iRSMD models, respectively. Distributions of probability score thresholds within the FNAT and iRMSD based top ranked prediction and the PatchDock based top solutions are plotted (Fig. 4A,B), which suggest docking score based ranked solutions are less likely to contain the native like complexes with respect to that achieved by FNAT and iRMSD based prediction. Out of the 320 interfaces (10 solutions for each complex), 12 interfaces were commonly predicted by FNAT and iRMSD models with highest reliability (probability threshold ≥ 0.95). ΔGs of binding for these predicted complexes are comparable with that achieved for known heterodimer complexes (Fig. 4C) indicating reliability of the predicted poses. Out of these 12 predicted complexes we showcase three complexes formed by glyceraldehyde 3-phosphate dehydrogenase (GAPDH) with phosphoglycerate kinase (PGK1), enolase 1 (ENO1) and triose-phosphate isomerase (TIM), respectively (Fig. 4D,F). Figure S6 provides the mode of interaction and the interface parameters for all the 12 complexes that were commonly predicted by FNAT and iRMSD models with highest reliability (probability threshold ≥ 0.95).

A web based server platform namely **P**rotein **C**omplex **P**rediction by **I**nterface **P**roperties (PCPIP) is developed to predict whether the interacting interface of a given protein–protein dimer complex resembles significantly with known protein interfaces. PCPIP predicts whether submitted interface(s) is likely to be native like or not. This prediction server would be particularly useful in identifying correct docking poses out of numerous solutions that standard protein docking programs offer. Figure S7 provides a snapshot of the input and output options of the PCPIP server.

## Discussion

Protein–protein interactions (PPI) are extremely crucial for intra and inter-cellular functions and inter-molecular connectivity. Due to significant improvement in experimental techniques, large numbers of protein structures are available now. Similarly, improved high-throughput studies like yeast two-hybrid system (Y2H), mass spectrometry (MS), tandem affinity purification (TAP) have identified numerous PPI that are previously unknown. However, these efforts are expensive, significantly time consuming, and have covered only a small portion of the complete PPI networks. Hence, the need for computational techniques has been increased to augment experimentally identified PPI and provide a larger repertoire of cellular PPIs. Another daunting challenge is to physically construct and map these large numbers of PPI complexes and identify the mode of interaction. Computationally generated PPI complexes could be quite useful and may expedite the experiments that are required to validate the binding interface and critical residues for the interactions. However, these predictions are generally error prone and therefore, need to be validated very carefully. Even though various protein–protein docking programs are available, methods for systematic evaluation of the predicted PPI complexes are limited.

In our effort, we tried to study the known protein–protein interface properties and utilize the knowledge of native PPI interface properties to a standard machine learning technique, support vector machine (SVM) to delineate native-like complexes from non-native like complexes. Interestingly, this simple approach turned out to be quite effective as suggested by very good performance metrics of our SVM models in distinguishing native and non-native like interfaces for homo and hetero complexes. Our exhaustive testing and benchmarking exercises using a completely non-redundant training–testing dataset and various degrees of distinguishing thresholds between native and non-native like complexes suggest a very high accuracy of the models. The categorizations of non-native instances were implemented using strict, moderate and lenient definitions to consider many intermediate docking models with partial overlap with native interfaces. Performance of the models demonstrate high efficiency of the approach in distinguishing native like complexes from non-native like complexes having high, moderate and low overlap with the actual interfaces (Table 1).

We also evaluated the performance of our method under further validation test where it was subjected to differentiate native like complexes from non-native complexes prepared from individually solved monomer structures of known complex structures. This apparently difficult dataset overrules the possibility of the monomers structures to be primed to form native complexes when subjected to protein docking approaches. In this Apo-Holo validation test, our method performs reasonably well, especially for the highly distinguished native and non-native like complexes (Table 2). However, comparative analysis using this dataset shows much better performance with respect to other available methods (Fig. 2).

We applied this approach in real scenario where protein interactions information is proven via experimental findings but the three-dimensional (3D) structure of the complexes and the subsequent interface(s) are yet to be discovered. We generated such complexes using 32 high-confidence STRING protein–protein interactions and identified the most likely interaction modes for 12 complexes. Such filtered structural models could be very useful for designing subsequent experiments to validate the actual mode of interaction even without attempting to solve the entire complex structures.

Finally, we converted the methodology into a user-friendly, easy-to-use web server platform namely PCPIP to predict whether the interacting interface of a given protein–protein dimer complex significantly resembles known protein interfaces. We believe that this resource could be a useful tool for biologists to evaluate protein–protein docking derived results and gain helpful knowledge to design confirmatory experiments.

## Supplementary information

Supplementary Information.
