# Supplementary material for: Classification and prediction of protein–protein interaction interface using machine learning algorithm
Source: Sci Rep. 2021 Jan 19;11:1761. doi: 10.1038/s41598-020-80900-2 (PMC7815773; doi:10.1038/s41598-020-80900-2)
Supplement: Supplementary file 1 — Supplementary Information. [file 41598_2020_80900_MOESM1_ESM.docx]

**Supplementary information file**

**Classification and prediction of protein-protein interaction interface using machine learning algorithm.**

**Subhrangshu Das and Saikat Chakrabarti***

**Contents**

**Collection and generation of protein-protein complex dataset 3**

**Generation of native and non-native like PPI complexes and their interfaces 3-4**

**Calculation of PPI interface properties 5**

**Performance measurement 5-6**

**Reference 6**

**Figure S1 7**

**Figure S2 8**

**Figure S3 9**

**Figure S4 10**

**Figure S5 11**

**Figure S6 12**

**Figure S7 13**

**Table S1 14**

**Table S2 15-25**

**Collection and generation of protein-protein complex dataset**

Initially, 989 protein-protein dimer complex structures have been derived from protein data bank (PDB) (1) using keyword based search with example keywords like homodimer or homo dimer, heterodimer or hetero dimer, etc., This way, we could obtain 560 homo and 429 hetero dimers. Exhaustive redundancy check and filter was applied to these datasets using the the CD-HIT (2) and BLASTp (3) programs so that no protein complex (both chains) is more than 40% identical to any other complex within the homo or hetero categories, respectively. CD-HIT was used to identify the sequence identity bases clusters whereas exhaustive pairwise alignment was performer using BLAST to identify the corresponding alignment coverage between any two pair of the proteins within our dataset. The complete list of the non-redundant set of homo and heterodimer list is available at http://www.hpppi.iicb.res.in/pcpip/download.php.

Validation dataset to perform the benchmarking were generated where both the dimer complex and individual monomer structures are available separately (Apo-Holo validation set). Complete list of these complexes are provided at http://www.hpppi.iicb.res.in/pcpip/download.php.

List of the STRING complexes are available at http://www.hpppi.iicb.res.in/pcpip/download.php.

We have also created a dataset of protein-protein complexes and subsequent interfaces for proteins that are not supposed to interact according to the Negatome database. We collected 130 such pair of non-interacting protein domains/chains available at http://www.hpppi.iicb.res.in/pcpip/download.php for which the 3D structures are available in PDB. After docking each pair, we collected 5 docked decoy complexes from the top, middle and last rank along with one each from the 1^st^ and 3^rd^ quartile of the solutions categorized based on PatchDock docking score.

**Generation of native and non-native like protein-protein complexes and their interfaces**

For a given dimer complex, monomer protein domains/chains were extracted out and were naively/blindly docked with each other using the PatchDock program. Native and non-native like complexes were identified from the docking solutions based on higher and lower degree of similarity of interacting interface defined by the following criteria.

*FNAT and iRMSD based categorization*

Critical Assessment of PRedicted Interactions (CAPRI) employed a standard complex evaluation criteria based on the fraction of conserved native contacts (FNAT) and the interface Root Mean Square Deviation (iRMSD). FNAT is the number of native (correct) residue–residue contacts in the docked (predicted) complex divided by the number of contacts in the original (known) complex whereas iRMSD is the root-mean-square deviation between the residues of both chain in the interface region. According to CAPRI criteria, predicted complexes with 0.1 ≤ FNAT < 0.3 and iRMSD ≤ 10 Å are regarded as acceptable predictions; 0.3 ≤ FNAT < 0.5 and iRMSD ≤ 5 Å as medium quality predictions; and FNAT ≥ 0.5 and iRMSD ≤ 1 Å as high quality predictions.

In our study, for homodimers and heterodimers, four sets of model were created keeping 1:5 ratio (2 native and 10 non-native samples, respectively) of native and non-native like complexes. All together, we could categorize 430 native and 2150 non-native like homodimer complexes in highly distinguishable, 360 native and 1800 non-native like homodimer complexes in moderately distinguishable, 416 native and 2080 non-native like homodimer complexes in weakly distinguishable, and 494 native and 2470 non-native like homodimer complexes in mixed distinguishable categories from 215, 180, 208, and 247 overlapping homodimer PDB entries, respectively. Similarly, we could categorize 406 native and 2030 non-native like heterodimer complexes in highly distinguishable, 290 native and 1450 non-native like heterodimer complexes in moderately distinguishable, 284 native and 1420 non-native like heterodimer complexes in weakly distinguishable, and 450 native and 2250 non-native like heterodimer complexes in mixed distinguishable categories from 203, 145, 142, and 225 overlapping heterodimer PDB entries, respectively (Table S1).

Similarly, native and non-native sets were also identified based on iRMSD. We have used the *Matchmaker* extension of Chimera program (4) to construct pairwise sequence alignments for superimposing the structures, which was further used to calculate iRMSD. One model each with 1:5 ratio (2 native and 10 non-native samples, respectively) of native and non-native like complexes were created for homodimer and heterodimer complexes. we could categorize 136 native and 680 non-native like homodimer complexes in highly distinguishable, 192 native and 960 non-native like homodimer complexes in moderately distinguishable, 232 native and 1160 non-native like homodimer complexes in weakly distinguishable, and 318 native and 1590 non-native like homodimer complexes in mixed distinguishable categories from 68, 96, 116, and 159 overlapping homodimer PDB entries, respectively. Similarly, we could categorize 318 native and 1590 non-native like heterodimer complexes in highly distinguishable, 500 native and 2500 non-native like heterodimer complexes in moderately distinguishable, 514 native and 2570 non-native like heterodimer complexes in weakly distinguishable, and 634 native and 3170 non-native like heterodimer complexes in mixed distinguishable categories from 159, 250, 257, and 317 overlapping heterodimer PDB entries, respectively (Table S1).

**Calculation of PPI interface properties**

In order to identify and subsequently analyze the properties of the interacting interfaces within the dimers, we used PISA, which calculates structural and chemical properties of macromolecular interfaces such as accessible/buried surface area, free energy of dissociation, presence/absence of hydrogen bond and salt bridges, etc.

*Energy of binding at the interface*

Strength of the binding at the interface is estimated via free energy of formation (ΔG_int_), solvation energy (SE) gain (ΔG_solv_) and the interface area. Details about these parameters are available at PISA documentations.

*Molecular interactions at the interface*

Interfaces are formed by residues from the participating protein subunits. Here, we have tried to find the pair of residues, one from each monomer, forming various types of molecular interactions, such as hydrogen bond and salt bridges. Interaction pairs for all possible combinations [190 cases ($C_{2}^{20}$)] of amino acid pair for each type of interactions were calculated.

*Solvent accessibility at the interface*

When two proteins come in contact to form an interacting interface, part of their surfaces becomes inaccessible to solvent. This is called buried surface area (BSA) whereas the part that remains accessible to solvent is called accessible surface area (ASA). BSA and ASA for each amino acid located at the interface were calculated from the PISA output.

In total, we have extracted 523 features which include binding energy, molecular interactions, accessible and buried surface area in the interface etc. for each complex. These features have been taken as input to train a support vector machine (SVM) to distinguish native-like complexes from non-native complexes.

**Performance measurement**

For both FNAT and iRMSD based categorizations, 100 fold randomized selection of the training (80% data) and testing (20% data) followed by SVM based classification trials were performed for each of the native and non-native complex threshold criterion. Figure S3 provides an overview of the various training and testing SVM runs employed in this study using multiple categories of native and non-native like protein-protein complexes. Our training model is able to predict a single entity with a probability threshold estimate of being native (T) or non-native (F) where, P (T) + P (F) = 1. The following parameters were calculated to measure the performance of our models in different scenario.

*Accuracy =*$\frac{TP+TN}{TP+TN+FP+FN}$ *Sensitivity or Recall or Native Positive Rate, TPR =* $\frac{TP}{TP+FN}$

*Specificity or Native Negative Rate, TNR =* $\frac{TN}{TN+FP}$

*NPV =* $\frac{TN}{TN+FN}$

*Precision =* $\frac{TP}{TP+FP}$

*F1 score (the harmonic mean of precision and sensitivity) =* $\frac{2TP}{2TP+FP+FN}$

*Matthews Correlation Coefficient (MCC) =* $\frac{TP x TN-FP x FN}{\sqrt{\left( TP+FP \right)\left( TP+FN \right)\left( TN+FP \right)\left( TN+FN \right)}}$

*Where TP = True Positive, TN = False Negative, FP = False Positive and FN = False Negative.*

**Reference:**

1. Berman HM, Westbrook J, Feng Z, Gilliland G, Bhat TN, Weissig H, Shindyalov IN, Bourne PE. The protein data bank. Nucleic acids research. 2000 Jan 1;28(1):235-42.

2. Fu L, Niu B, Zhu Z, Wu S, Li W. CD-HIT: accelerated for clustering the next-generation sequencing data. Bioinformatics. 2012 Dec 1;28(23):3150-2.

3. Altschul SF, Madden TL, Schäffer AA, Zhang J, Zhang Z, Miller W, Lipman DJ. Gapped BLAST and PSI-BLAST: a new generation of protein database search programs. Nucleic acids research. 1997 Sep 1;25(17):3389-402.

4. Pettersen EF, Goddard TD, Huang CC, Couch GS, Greenblatt DM, Meng EC, Ferrin TE.UCSF Chimera--a visualization system for exploratory research and analysis J Comput Chem. 2004 Oct;25(13):1605-12.

**Figure S1**


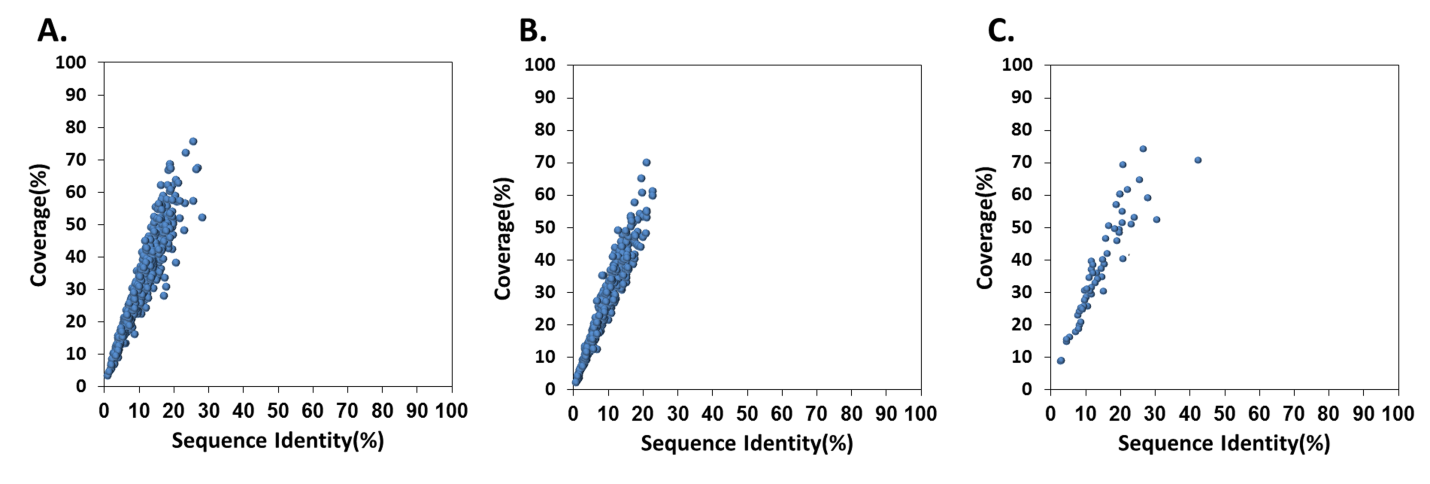


**Figure S1:** Sequence identity of the benchmarking proteins with respect to the proteins used in classification models. Highest sequence coverage (Y axis) and identity (X axis) were calculated for each of the protein in the homo (A) and hetero (B) and Apo-Holo (C) benchmarking sets with respect to their corresponding SVM model dataset.

**Figure S2**


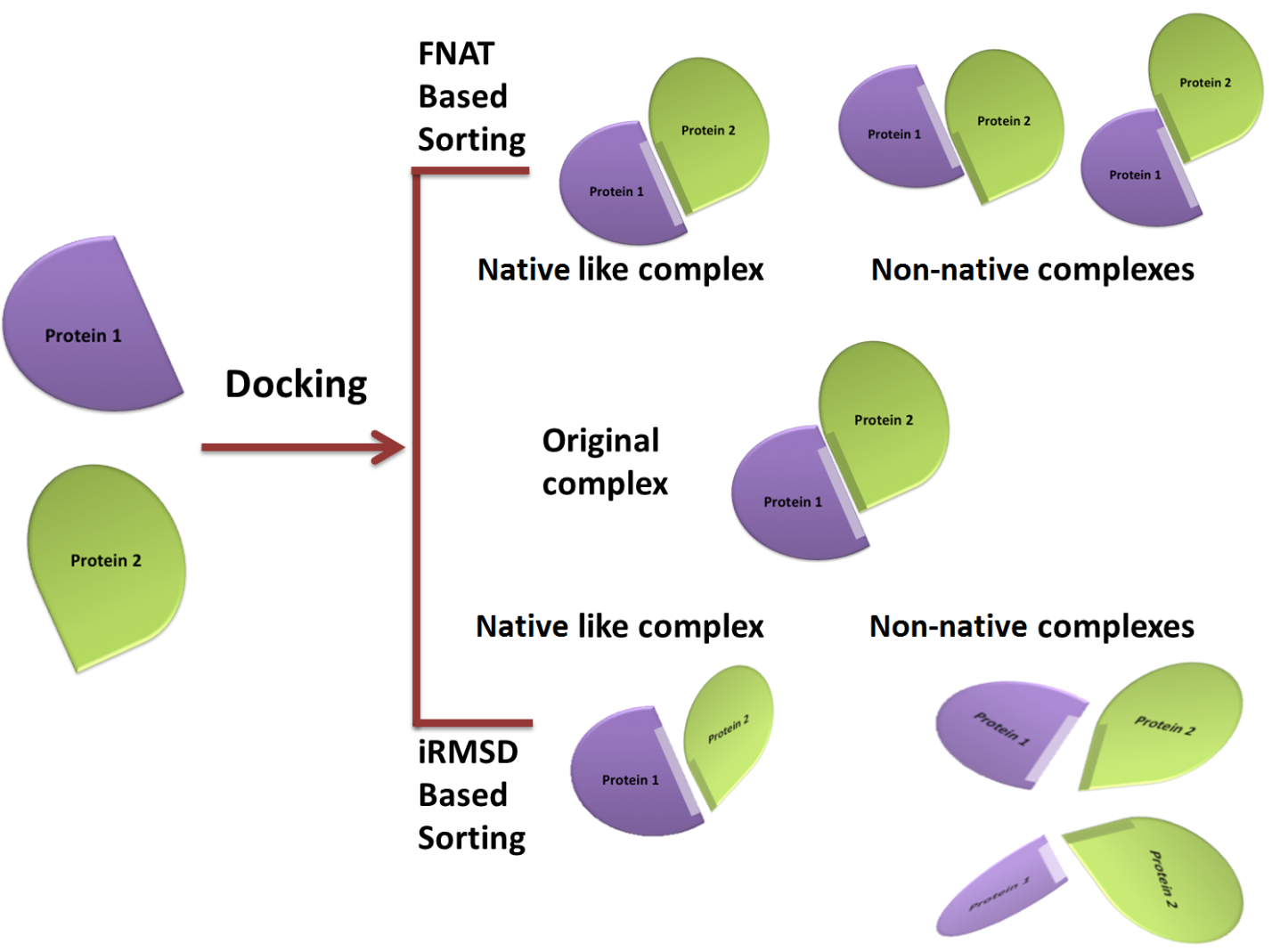


**Figure S2:** Schematic representation of the approach of generating native and non-native like protein-protein complexes and their interfaces.

**Figure S3**


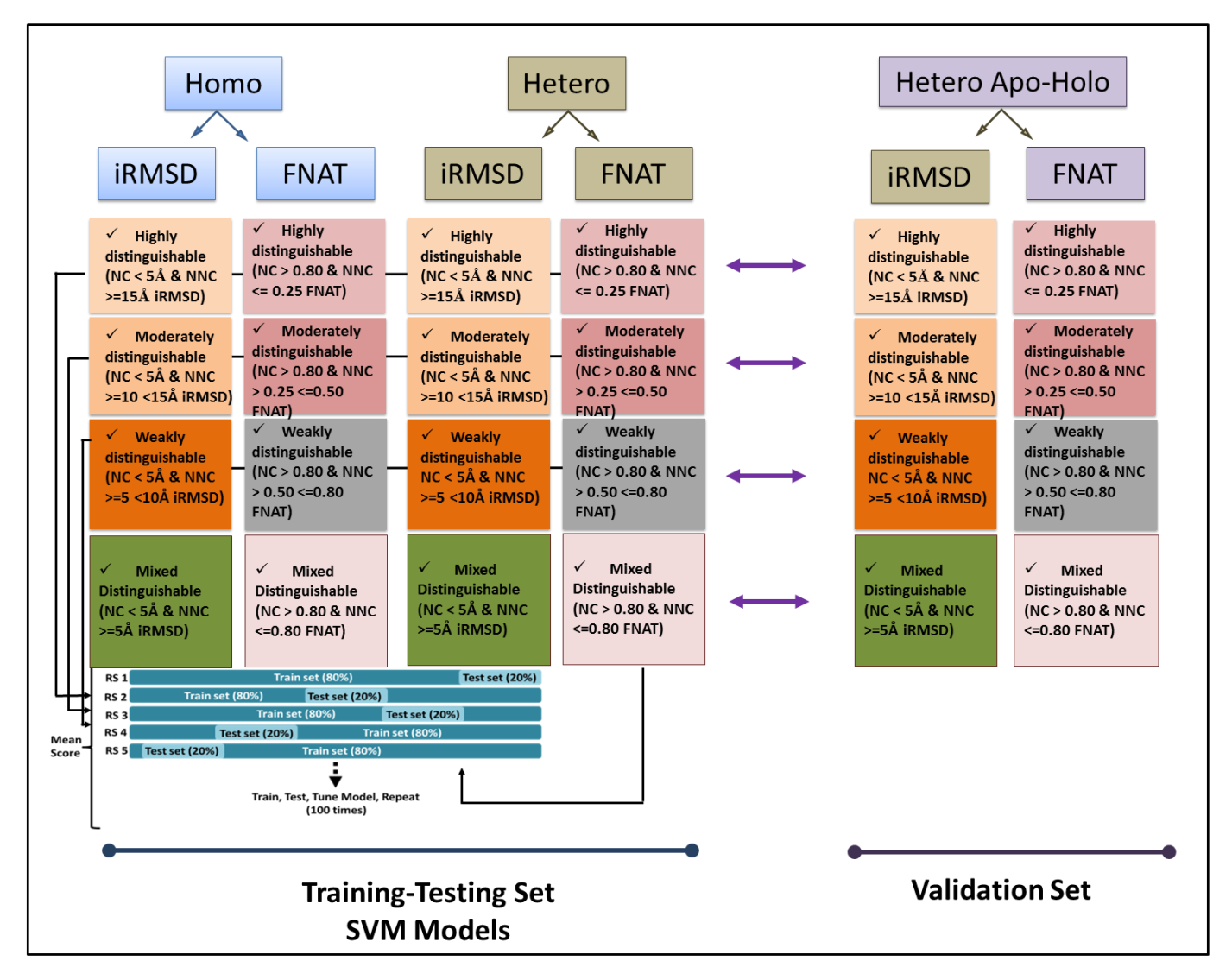


**Figure S3:** Dataset and benchmarking protocol.

**Figure S4**


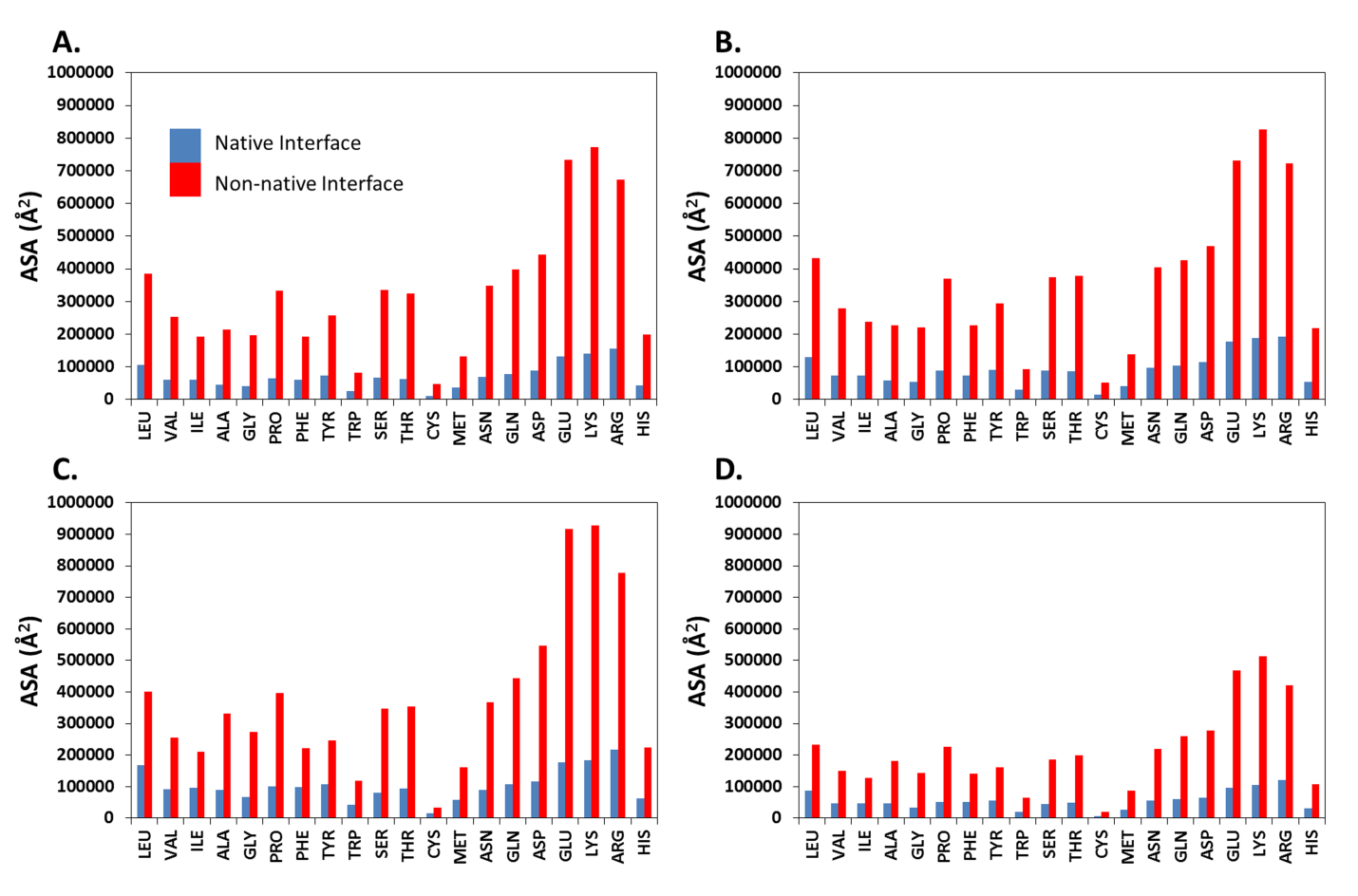


**Figure S4:** Average accessible surface area (ASA) of amino acids located at the native and non-native like interfaces. Average ASAs of amino acids at the native and non-native interfaces identified based on FNAT (left panels) and iRMSD (right panels) definition for hetero (A), and homo (B) complexes, respectively.

**Figure S5**


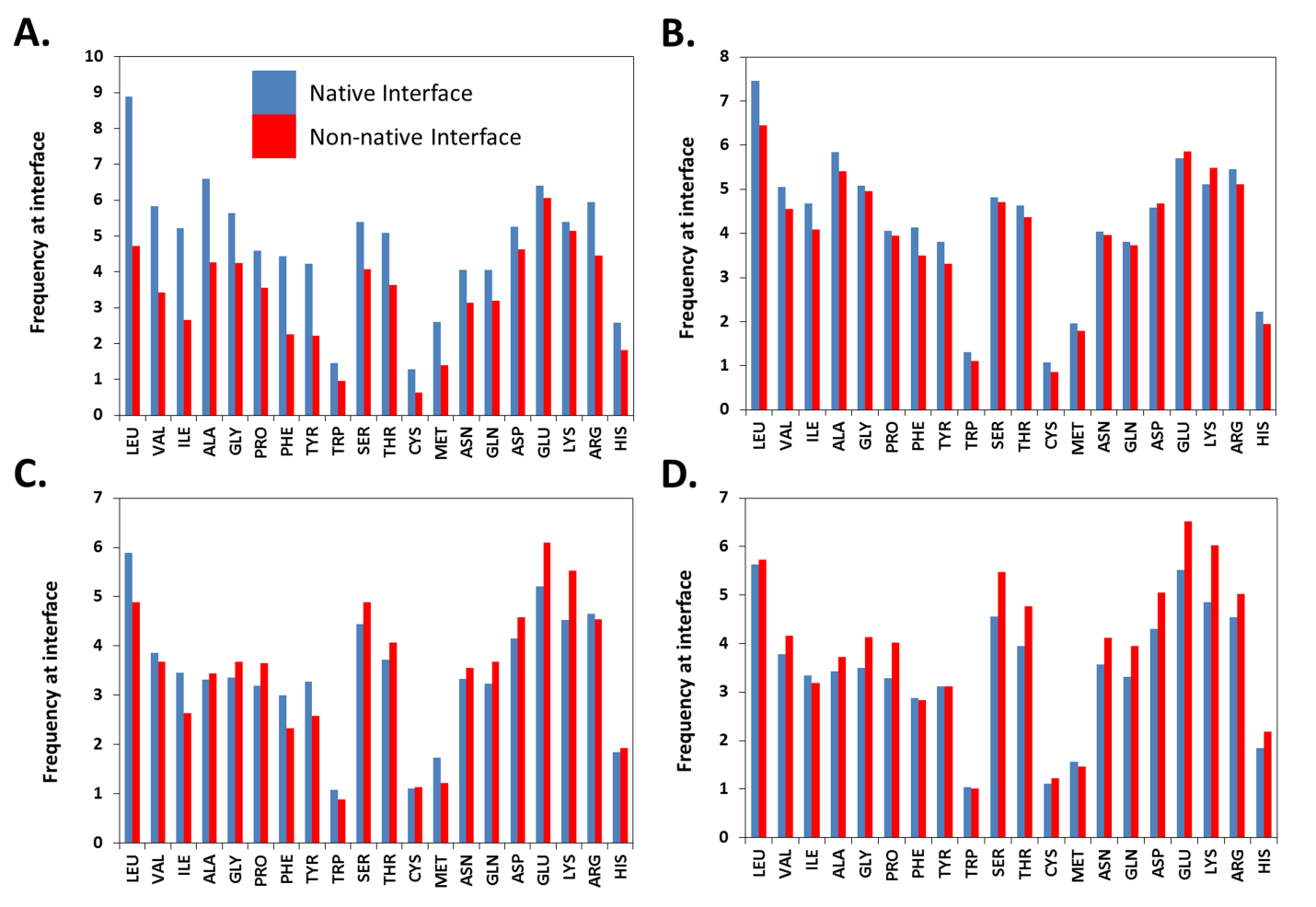


**Figure S5:** Frequency of amino acids at the native and non-native like interfaces. Average frequency of each amino acid located at the native and non-native like interfaces is plotted for homo (A: FNAT; B: iRMSD) and hetero (C: FNAT; D: iRMSD) complexes, respectively.

**Figure S6**


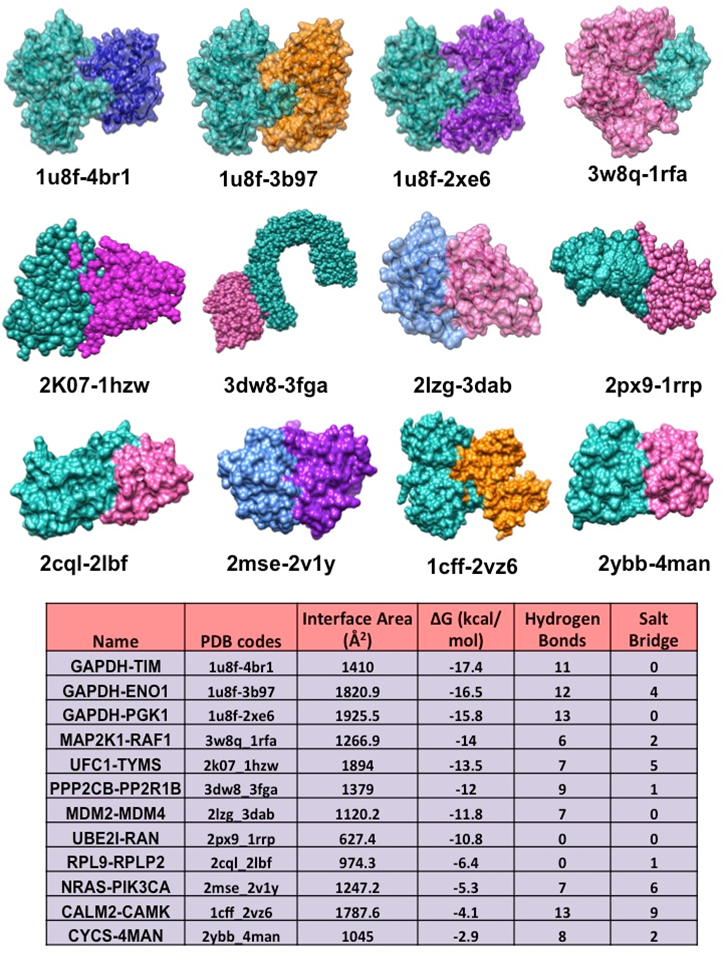


**Figure S6:** Structural details of the predicted hetero complexes collected from STRING database. Mode of interaction and the predicted interface parameters for the 12 complexes that were commonly predicted by FNAT and iRMSD models with highest reliability (probability threshold >= 0.95). These are known and proven human protein-protein interactions reported in STRING database for which complex structure is not solved.

**Figure S7**


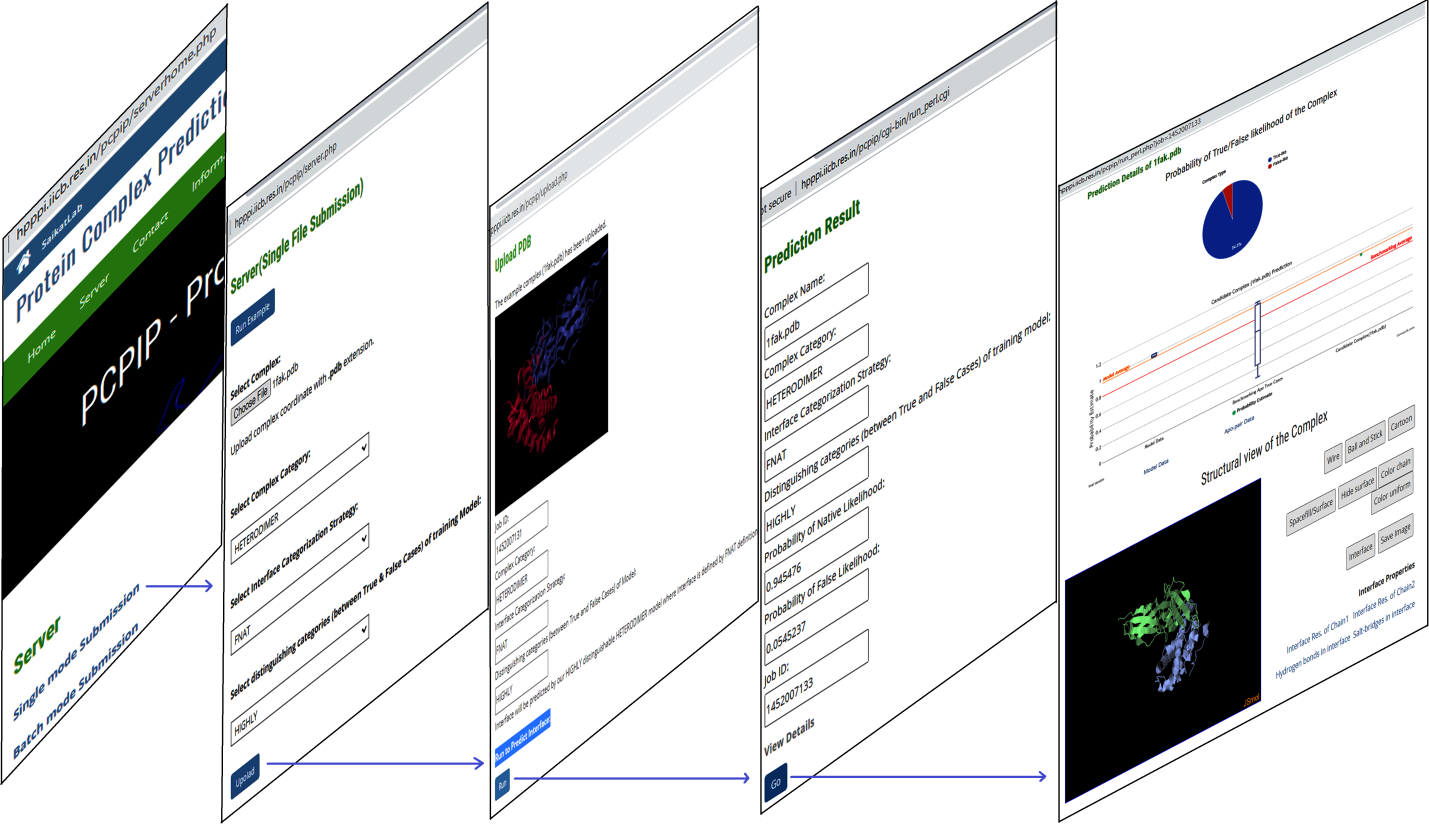


**Figure S7:** Snapshot of the input and output options of the PCPIP server.

**Table S1: Training, testing, benchmarking and validation datasets.**

| **PPI category** | **SVM model** | | **SVM model** | | **Apo-Holo validation set** | | **Apo-Holo validation set** | | |
| --- | --- | --- | --- | --- | --- | --- | --- | --- | --- |
|  | **(FNAT)** | | **(iRMSD)** | | **(FNAT)** | | **(iRMSD)** | | |
|  | **Dimer** | **Samples** | **Dimer** | **Samples** | **Dimer** | **Samples** | **Dimer** | | **Samples** |
| Homo | 215 | Native(**FNAT>80**): 430 | 68 | Native(**IRMSD<5Å**): 136 |  | |  | | |
|  |  | Non-native(**FNAT≤25**): 2150 |  | Non-native(**IRMSD≥15Å**): 680 |  |  |  | | |
|  | 180 | Native(**FNAT>80**): 360 | 96 | Native(**IRMSD<5Å**): 192 |  |  |  | | |
|  |  | Non-native(**FNAT>25 & ≤50**): 1800 |  | Non-native(**IRMSD≥10Å & <15Å**): 960 |  | |  | | |
|  | 208 | Native(**FNAT>80**): 416 | 116 | Native(**IRMSD<5Å**): 232 |  | |  | | |
|  |  | Non-native(**FNAT>50 & ≤80**): 2080 |  | Non-native(**IRMSD≥5Å & <10Å**): 1160 |  |  |  | | |
|  | 247 | Native(**FNAT>80**): 494 | 159 | Native(**IRMSD<5Å**): 318 |  |  |  | | |
|  |  | Non-native(**FNAT≤80**): 2470 |  | Non-native(**IRMSD≥5Å**): 1590 |  |  |  | | |
| Hetero | 203 | Native(**FNAT>80**): 406 | 159 | Native(**IRMSD<5Å**): 318 | 28 | Native(**FNAT>80**): 56 | 49 | Native(**IRMSD<5Å**): 98 | |
|  |  | Non-native(**FNAT≤25**): 2030 |  | Non-native(**IRMSD≥15Å**): 1590 |  | Non-native(**FNAT≤25**): 280 |  | Non-native(**IRMSD≥15Å**): 490 | |
|  | 145 | Native(**FNAT>80**): 290 | 250 | Native(**IRMSD<5Å**): 500 | 5 | Native(**FNAT>80**): 10 | 68 | Native(**IRMSD<5Å**): 134 | |
|  |  | Non-native(**FNAT>25 & ≤50**): 1450 |  | Non-native(**IRMSD≥10Å & <15Å**): 2500 |  | Non-native(**FNAT>25 & ≤50**): 50 |  | Non-native(**IRMSD≥10Å & <15Å**): 680 | |
|  | 142 | Native(**FNAT>80**): 284 | 257 | Native(**IRMSD<5Å**): 514 | 10 | Native(**FNAT>80**): 20 | 68 | Native(**IRMSD<5Å**): 134 | |
|  |  | Non-native(**FNAT>50 & ≤80**): 1420 |  | Non-native(**IRMSD≥5Å & <10Å**): 2570 |  | Non-native(**FNAT>50 & ≤80**): 100 |  | Non-native(**IRMSD≥5Å & <10Å**): 680 | |
|  | 225 | Native(**FNAT>80**): 450 | 317 | Native(**IRMSD<5Å**): 634 | 32 | Native(**FNAT>80**): 64 | 68 | Native(**IRMSD<5Å**): 134 | |
|  |  | Non-native(**FNAT≤80**): 2250 |  | Non-native(**IRMSD≥5Å**): 3170 |  | Non-native(**FNAT≤80**): 320 |  | Non-native(**IRMSD≥5Å**): 680 | |

**# Native and Non-native samples were created by FNAT and iRMSD based definition; Dimer: Number of original complexes;**

**Samples: Native and Non-native data; Native: Native like complexes; Non-native: Non-native like complexes;**

**Table S2: List of all and common distinguishable protein-protein interaction interface features.**

| **Serial No.** | **Features** |
| --- | --- |
| 1 | Binding Energy (Delta G)* |
| 2 | Frequency of Hydrogen Bond formed between LEU-LEU |
| 3 | Frequency of Hydrogen Bond formed between LEU-VAL |
| 4 | Frequency of Hydrogen Bond formed between LEU-ILE |
| 5 | Frequency of Hydrogen Bond formed between LEU-ALA |
| 6 | Frequency of Hydrogen Bond formed between LEU-GLY |
| 7 | Frequency of Hydrogen Bond formed between LEU-PRO |
| 8 | Frequency of Hydrogen Bond formed between LEU-PHE |
| 9 | Frequency of Hydrogen Bond formed between LEU-TYR* |
| 10 | Frequency of Hydrogen Bond formed between LEU-TRP |
| 11 | Frequency of Hydrogen Bond formed between LEU-SER |
| 12 | Frequency of Hydrogen Bond formed between LEU-THR |
| 13 | Frequency of Hydrogen Bond formed between LEU-CYS |
| 14 | Frequency of Hydrogen Bond formed between LEU-MET |
| 15 | Frequency of Hydrogen Bond formed between LEU-ASN |
| 16 | Frequency of Hydrogen Bond formed between LEU-GLN |
| 17 | Frequency of Hydrogen Bond formed between LEU-ASP |
| 18 | Frequency of Hydrogen Bond formed between LEU-GLU |
| 19 | Frequency of Hydrogen Bond formed between LEU-LYS |
| 20 | Frequency of Hydrogen Bond formed between LEU-ARG* |
| 21 | Frequency of Hydrogen Bond formed between LEU-HIS |
| 22 | Frequency of Hydrogen Bond formed between VAL-VAL |
| 23 | Frequency of Hydrogen Bond formed between VAL-ILE |
| 24 | Frequency of Hydrogen Bond formed between VAL-ALA |
| 25 | Frequency of Hydrogen Bond formed between VAL-GLY |
| 26 | Frequency of Hydrogen Bond formed between VAL-PRO |
| 27 | Frequency of Hydrogen Bond formed between VAL-PHE |
| 28 | Frequency of Hydrogen Bond formed between VAL-TYR |
| 29 | Frequency of Hydrogen Bond formed between VAL-TRP |
| 30 | Frequency of Hydrogen Bond formed between VAL-SER |
| 31 | Frequency of Hydrogen Bond formed between VAL-THR |
| 32 | Frequency of Hydrogen Bond formed between VAL-CYS |
| 33 | Frequency of Hydrogen Bond formed between VAL-MET |
| 34 | Frequency of Hydrogen Bond formed between VAL-ASN |
| 35 | Frequency of Hydrogen Bond formed between VAL-GLN |
| 36 | Frequency of Hydrogen Bond formed between VAL-ASP |
| 37 | Frequency of Hydrogen Bond formed between VAL-GLU |
| 38 | Frequency of Hydrogen Bond formed between VAL-LYS |
| 39 | Frequency of Hydrogen Bond formed between VAL-ARG |
| 40 | Frequency of Hydrogen Bond formed between VAL-HIS |
| 41 | Frequency of Hydrogen Bond formed between ILE-ILE |
| 42 | Frequency of Hydrogen Bond formed between ILE-ALA |
| 43 | Frequency of Hydrogen Bond formed between ILE-GLY |
| 44 | Frequency of Hydrogen Bond formed between ILE-PRO |
| 45 | Frequency of Hydrogen Bond formed between ILE-PHE |
| 46 | Frequency of Hydrogen Bond formed between ILE-TYR |
| 47 | Frequency of Hydrogen Bond formed between ILE-TRP |
| 48 | Frequency of Hydrogen Bond formed between ILE-SER |
| 49 | Frequency of Hydrogen Bond formed between ILE-THR |
| 50 | Frequency of Hydrogen Bond formed between ILE-CYS |
| 51 | Frequency of Hydrogen Bond formed between ILE-MET |
| 52 | Frequency of Hydrogen Bond formed between ILE-ASN |
| 53 | Frequency of Hydrogen Bond formed between ILE-GLN |
| 54 | Frequency of Hydrogen Bond formed between ILE-ASP |
| 55 | Frequency of Hydrogen Bond formed between ILE-GLU |
| 56 | Frequency of Hydrogen Bond formed between ILE-LYS |
| 57 | Frequency of Hydrogen Bond formed between ILE-ARG |
| 58 | Frequency of Hydrogen Bond formed between ILE-HIS |
| 59 | Frequency of Hydrogen Bond formed between ALA-ALA |
| 60 | Frequency of Hydrogen Bond formed between ALA-GLY |
| 61 | Frequency of Hydrogen Bond formed between ALA-PRO |
| 62 | Frequency of Hydrogen Bond formed between ALA-PHE |
| 63 | Frequency of Hydrogen Bond formed between ALA-TYR |
| 64 | Frequency of Hydrogen Bond formed between ALA-TRP |
| 65 | Frequency of Hydrogen Bond formed between ALA-SER |
| 66 | Frequency of Hydrogen Bond formed between ALA-THR |
| 67 | Frequency of Hydrogen Bond formed between ALA-CYS |
| 68 | Frequency of Hydrogen Bond formed between ALA-MET |
| 69 | Frequency of Hydrogen Bond formed between ALA-ASN |
| 70 | Frequency of Hydrogen Bond formed between ALA-GLN |
| 71 | Frequency of Hydrogen Bond formed between ALA-ASP |
| 72 | Frequency of Hydrogen Bond formed between ALA-GLU |
| 73 | Frequency of Hydrogen Bond formed between ALA-LYS |
| 74 | Frequency of Hydrogen Bond formed between ALA-ARG |
| 75 | Frequency of Hydrogen Bond formed between ALA-HIS |
| 76 | Frequency of Hydrogen Bond formed between GLY-GLY |
| 77 | Frequency of Hydrogen Bond formed between GLY-PRO |
| 78 | Frequency of Hydrogen Bond formed between GLY-PHE |
| 79 | Frequency of Hydrogen Bond formed between GLY-TYR |
| 80 | Frequency of Hydrogen Bond formed between GLY-TRP |
| 81 | Frequency of Hydrogen Bond formed between GLY-SER |
| 82 | Frequency of Hydrogen Bond formed between GLY-THR |
| 83 | Frequency of Hydrogen Bond formed between GLY-CYS |
| 84 | Frequency of Hydrogen Bond formed between GLY-MET |
| 85 | Frequency of Hydrogen Bond formed between GLY-ASN |
| 86 | Frequency of Hydrogen Bond formed between GLY-GLN |
| 87 | Frequency of Hydrogen Bond formed between GLY-ASP |
| 88 | Frequency of Hydrogen Bond formed between GLY-GLU |
| 89 | Frequency of Hydrogen Bond formed between GLY-LYS |
| 90 | Frequency of Hydrogen Bond formed between GLY-ARG* |
| 91 | Frequency of Hydrogen Bond formed between GLY-HIS |
| 92 | Frequency of Hydrogen Bond formed between PRO-PRO |
| 93 | Frequency of Hydrogen Bond formed between PRO-PHE |
| 94 | Frequency of Hydrogen Bond formed between PRO-TYR |
| 95 | Frequency of Hydrogen Bond formed between PRO-TRP |
| 96 | Frequency of Hydrogen Bond formed between PRO-SER |
| 97 | Frequency of Hydrogen Bond formed between PRO-THR |
| 98 | Frequency of Hydrogen Bond formed between PRO-CYS |
| 99 | Frequency of Hydrogen Bond formed between PRO-MET |
| 100 | Frequency of Hydrogen Bond formed between PRO-ASN |
| 101 | Frequency of Hydrogen Bond formed between PRO-GLN |
| 102 | Frequency of Hydrogen Bond formed between PRO-ASP |
| 103 | Frequency of Hydrogen Bond formed between PRO-GLU |
| 104 | Frequency of Hydrogen Bond formed between PRO-LYS |
| 105 | Frequency of Hydrogen Bond formed between PRO-ARG |
| 106 | Frequency of Hydrogen Bond formed between PRO-HIS |
| 107 | Frequency of Hydrogen Bond formed between PHE-PHE |
| 108 | Frequency of Hydrogen Bond formed between PHE-TYR |
| 109 | Frequency of Hydrogen Bond formed between PHE-TRP |
| 110 | Frequency of Hydrogen Bond formed between PHE-SER |
| 111 | Frequency of Hydrogen Bond formed between PHE-THR |
| 112 | Frequency of Hydrogen Bond formed between PHE-CYS |
| 113 | Frequency of Hydrogen Bond formed between PHE-MET |
| 114 | Frequency of Hydrogen Bond formed between PHE-ASN |
| 115 | Frequency of Hydrogen Bond formed between PHE-GLN |
| 116 | Frequency of Hydrogen Bond formed between PHE-ASP |
| 117 | Frequency of Hydrogen Bond formed between PHE-GLU |
| 118 | Frequency of Hydrogen Bond formed between PHE-LYS |
| 119 | Frequency of Hydrogen Bond formed between PHE-ARG |
| 120 | Frequency of Hydrogen Bond formed between PHE-HIS |
| 121 | Frequency of Hydrogen Bond formed between TYR-TYR |
| 122 | Frequency of Hydrogen Bond formed between TYR-TRP |
| 123 | Frequency of Hydrogen Bond formed between TYR-SER |
| 124 | Frequency of Hydrogen Bond formed between TYR-THR* |
| 125 | Frequency of Hydrogen Bond formed between TYR-CYS |
| 126 | Frequency of Hydrogen Bond formed between TYR-MET |
| 127 | Frequency of Hydrogen Bond formed between TYR-ASN |
| 128 | Frequency of Hydrogen Bond formed between TYR-GLN |
| 129 | Frequency of Hydrogen Bond formed between TYR-ASP |
| 130 | Frequency of Hydrogen Bond formed between TYR-GLU |
| 131 | Frequency of Hydrogen Bond formed between TYR-LYS |
| 132 | Frequency of Hydrogen Bond formed between TYR-ARG* |
| 133 | Frequency of Hydrogen Bond formed between TYR-HIS |
| 134 | Frequency of Hydrogen Bond formed between TRP-TRP |
| 135 | Frequency of Hydrogen Bond formed between TRP-SER |
| 136 | Frequency of Hydrogen Bond formed between TRP-THR |
| 137 | Frequency of Hydrogen Bond formed between TRP-CYS |
| 138 | Frequency of Hydrogen Bond formed between TRP-MET |
| 139 | Frequency of Hydrogen Bond formed between TRP-ASN |
| 140 | Frequency of Hydrogen Bond formed between TRP-GLN |
| 141 | Frequency of Hydrogen Bond formed between TRP-ASP |
| 142 | Frequency of Hydrogen Bond formed between TRP-GLU |
| 143 | Frequency of Hydrogen Bond formed between TRP-LYS |
| 144 | Frequency of Hydrogen Bond formed between TRP-ARG |
| 145 | Frequency of Hydrogen Bond formed between TRP-HIS |
| 146 | Frequency of Hydrogen Bond formed between SER-SER |
| 147 | Frequency of Hydrogen Bond formed between SER-THR |
| 148 | Frequency of Hydrogen Bond formed between SER-CYS |
| 149 | Frequency of Hydrogen Bond formed between SER-MET |
| 150 | Frequency of Hydrogen Bond formed between SER-ASN |
| 151 | Frequency of Hydrogen Bond formed between SER-GLN |
| 152 | Frequency of Hydrogen Bond formed between SER-ASP |
| 153 | Frequency of Hydrogen Bond formed between SER-GLU |
| 154 | Frequency of Hydrogen Bond formed between SER-LYS |
| 155 | Frequency of Hydrogen Bond formed between SER-ARG |
| 156 | Frequency of Hydrogen Bond formed between SER-HIS |
| 157 | Frequency of Hydrogen Bond formed between THR-THR |
| 158 | Frequency of Hydrogen Bond formed between THR-CYS |
| 159 | Frequency of Hydrogen Bond formed between THR-MET |
| 160 | Frequency of Hydrogen Bond formed between THR-ASN |
| 161 | Frequency of Hydrogen Bond formed between THR-GLN |
| 162 | Frequency of Hydrogen Bond formed between THR-ASP |
| 163 | Frequency of Hydrogen Bond formed between THR-GLU |
| 164 | Frequency of Hydrogen Bond formed between THR-LYS |
| 165 | Frequency of Hydrogen Bond formed between THR-ARG* |
| 166 | Frequency of Hydrogen Bond formed between THR-HIS |
| 167 | Frequency of Hydrogen Bond formed between CYS-CYS |
| 168 | Frequency of Hydrogen Bond formed between CYS-MET |
| 169 | Frequency of Hydrogen Bond formed between CYS-ASN |
| 170 | Frequency of Hydrogen Bond formed between CYS-GLN |
| 171 | Frequency of Hydrogen Bond formed between CYS-ASP |
| 172 | Frequency of Hydrogen Bond formed between CYS-GLU |
| 173 | Frequency of Hydrogen Bond formed between CYS-LYS |
| 174 | Frequency of Hydrogen Bond formed between CYS-ARG |
| 175 | Frequency of Hydrogen Bond formed between CYS-HIS |
| 176 | Frequency of Hydrogen Bond formed between MET-MET |
| 177 | Frequency of Hydrogen Bond formed between MET-ASN |
| 178 | Frequency of Hydrogen Bond formed between MET-GLN |
| 179 | Frequency of Hydrogen Bond formed between MET-ASP |
| 180 | Frequency of Hydrogen Bond formed between MET-GLU |
| 181 | Frequency of Hydrogen Bond formed between MET-LYS |
| 182 | Frequency of Hydrogen Bond formed between MET-ARG |
| 183 | Frequency of Hydrogen Bond formed between MET-HIS |
| 184 | Frequency of Hydrogen Bond formed between ASN-ASN |
| 185 | Frequency of Hydrogen Bond formed between ASN-GLN |
| 186 | Frequency of Hydrogen Bond formed between ASN-ASP |
| 187 | Frequency of Hydrogen Bond formed between ASN-GLU |
| 188 | Frequency of Hydrogen Bond formed between ASN-LYS |
| 189 | Frequency of Hydrogen Bond formed between ASN-ARG |
| 190 | Frequency of Hydrogen Bond formed between ASN-HIS |
| 191 | Frequency of Hydrogen Bond formed between GLN-GLN* |
| 192 | Frequency of Hydrogen Bond formed between GLN-ASP |
| 193 | Frequency of Hydrogen Bond formed between GLN-GLU |
| 194 | Frequency of Hydrogen Bond formed between GLN-LYS |
| 195 | Frequency of Hydrogen Bond formed between GLN-ARG |
| 196 | Frequency of Hydrogen Bond formed between GLN-HIS |
| 197 | Frequency of Hydrogen Bond formed between ASP-ASP |
| 198 | Frequency of Hydrogen Bond formed between ASP-GLU |
| 199 | Frequency of Hydrogen Bond formed between ASP-LYS |
| 200 | Frequency of Hydrogen Bond formed between ASP-ARG* |
| 201 | Frequency of Hydrogen Bond formed between ASP-HIS |
| 202 | Frequency of Hydrogen Bond formed between GLU-GLU |
| 203 | Frequency of Hydrogen Bond formed between GLU-LYS |
| 204 | Frequency of Hydrogen Bond formed between GLU-ARG* |
| 205 | Frequency of Hydrogen Bond formed between GLU-HIS |
| 206 | Frequency of Hydrogen Bond formed between LYS-LYS |
| 207 | Frequency of Hydrogen Bond formed between LYS-ARG |
| 208 | Frequency of Hydrogen Bond formed between LYS-HIS |
| 209 | Frequency of Hydrogen Bond formed between ARG-ARG |
| 210 | Frequency of Hydrogen Bond formed between ARG-HIS |
| 211 | Frequency of Hydrogen Bond formed between HIS-HIS |
| 212 | Average Distance of Hydrogen Bond |
| 213 | Frequency of Salt-bridge formed between LEU-LEU |
| 214 | Frequency of Salt-bridge formed between LEU-VAL |
| 215 | Frequency of Salt-bridge formed between LEU-ILE |
| 216 | Frequency of Salt-bridge formed between LEU-ALA |
| 217 | Frequency of Salt-bridge formed between LEU-GLY |
| 218 | Frequency of Salt-bridge formed between LEU-PRO |
| 219 | Frequency of Salt-bridge formed between LEU-PHE |
| 220 | Frequency of Salt-bridge formed between LEU-TYR |
| 221 | Frequency of Salt-bridge formed between LEU-TRP |
| 222 | Frequency of Salt-bridge formed between LEU-SER |
| 223 | Frequency of Salt-bridge formed between LEU-THR |
| 224 | Frequency of Salt-bridge formed between LEU-CYS |
| 225 | Frequency of Salt-bridge formed between LEU-MET |
| 226 | Frequency of Salt-bridge formed between LEU-ASN |
| 227 | Frequency of Salt-bridge formed between LEU-GLN |
| 228 | Frequency of Salt-bridge formed between LEU-ASP |
| 229 | Frequency of Salt-bridge formed between LEU-GLU |
| 230 | Frequency of Salt-bridge formed between LEU-LYS |
| 231 | Frequency of Salt-bridge formed between LEU-ARG |
| 232 | Frequency of Salt-bridge formed between LEU-HIS |
| 233 | Frequency of Salt-bridge formed between VAL-VAL |
| 234 | Frequency of Salt-bridge formed between VAL-ILE |
| 235 | Frequency of Salt-bridge formed between VAL-ALA |
| 236 | Frequency of Salt-bridge formed between VAL-GLY |
| 237 | Frequency of Salt-bridge formed between VAL-PRO |
| 238 | Frequency of Salt-bridge formed between VAL-PHE |
| 239 | Frequency of Salt-bridge formed between VAL-TYR |
| 240 | Frequency of Salt-bridge formed between VAL-TRP |
| 241 | Frequency of Salt-bridge formed between VAL-SER |
| 242 | Frequency of Salt-bridge formed between VAL-THR |
| 243 | Frequency of Salt-bridge formed between VAL-CYS |
| 244 | Frequency of Salt-bridge formed between VAL-MET |
| 245 | Frequency of Salt-bridge formed between VAL-ASN |
| 246 | Frequency of Salt-bridge formed between VAL-GLN |
| 247 | Frequency of Salt-bridge formed between VAL-ASP |
| 248 | Frequency of Salt-bridge formed between VAL-GLU |
| 249 | Frequency of Salt-bridge formed between VAL-LYS |
| 250 | Frequency of Salt-bridge formed between VAL-ARG |
| 251 | Frequency of Salt-bridge formed between VAL-HIS |
| 252 | Frequency of Salt-bridge formed between ILE-ILE |
| 253 | Frequency of Salt-bridge formed between ILE-ALA |
| 254 | Frequency of Salt-bridge formed between ILE-GLY |
| 255 | Frequency of Salt-bridge formed between ILE-PRO |
| 256 | Frequency of Salt-bridge formed between ILE-PHE |
| 257 | Frequency of Salt-bridge formed between ILE-TYR |
| 258 | Frequency of Salt-bridge formed between ILE-TRP |
| 259 | Frequency of Salt-bridge formed between ILE-SER |
| 260 | Frequency of Salt-bridge formed between ILE-THR |
| 261 | Frequency of Salt-bridge formed between ILE-CYS |
| 262 | Frequency of Salt-bridge formed between ILE-MET |
| 263 | Frequency of Salt-bridge formed between ILE-ASN |
| 264 | Frequency of Salt-bridge formed between ILE-GLN |
| 265 | Frequency of Salt-bridge formed between ILE-ASP |
| 266 | Frequency of Salt-bridge formed between ILE-GLU |
| 267 | Frequency of Salt-bridge formed between ILE-LYS |
| 268 | Frequency of Salt-bridge formed between ILE-ARG |
| 269 | Frequency of Salt-bridge formed between ILE-HIS |
| 270 | Frequency of Salt-bridge formed between ALA-ALA |
| 271 | Frequency of Salt-bridge formed between ALA-GLY |
| 272 | Frequency of Salt-bridge formed between ALA-PRO |
| 273 | Frequency of Salt-bridge formed between ALA-PHE |
| 274 | Frequency of Salt-bridge formed between ALA-TYR |
| 275 | Frequency of Salt-bridge formed between ALA-TRP |
| 276 | Frequency of Salt-bridge formed between ALA-SER |
| 277 | Frequency of Salt-bridge formed between ALA-THR |
| 278 | Frequency of Salt-bridge formed between ALA-CYS |
| 279 | Frequency of Salt-bridge formed between ALA-MET |
| 280 | Frequency of Salt-bridge formed between ALA-ASN |
| 281 | Frequency of Salt-bridge formed between ALA-GLN |
| 282 | Frequency of Salt-bridge formed between ALA-ASP |
| 283 | Frequency of Salt-bridge formed between ALA-GLU |
| 284 | Frequency of Salt-bridge formed between ALA-LYS |
| 285 | Frequency of Salt-bridge formed between ALA-ARG |
| 286 | Frequency of Salt-bridge formed between ALA-HIS |
| 287 | Frequency of Salt-bridge formed between GLY-GLY |
| 288 | Frequency of Salt-bridge formed between GLY-PRO |
| 289 | Frequency of Salt-bridge formed between GLY-PHE |
| 290 | Frequency of Salt-bridge formed between GLY-TYR |
| 291 | Frequency of Salt-bridge formed between GLY-TRP |
| 292 | Frequency of Salt-bridge formed between GLY-SER |
| 293 | Frequency of Salt-bridge formed between GLY-THR |
| 294 | Frequency of Salt-bridge formed between GLY-CYS |
| 295 | Frequency of Salt-bridge formed between GLY-MET |
| 296 | Frequency of Salt-bridge formed between GLY-ASN |
| 297 | Frequency of Salt-bridge formed between GLY-GLN |
| 298 | Frequency of Salt-bridge formed between GLY-ASP |
| 299 | Frequency of Salt-bridge formed between GLY-GLU |
| 300 | Frequency of Salt-bridge formed between GLY-LYS |
| 301 | Frequency of Salt-bridge formed between GLY-ARG |
| 302 | Frequency of Salt-bridge formed between GLY-HIS |
| 303 | Frequency of Salt-bridge formed between PRO-PRO |
| 304 | Frequency of Salt-bridge formed between PRO-PHE |
| 305 | Frequency of Salt-bridge formed between PRO-TYR |
| 306 | Frequency of Salt-bridge formed between PRO-TRP |
| 307 | Frequency of Salt-bridge formed between PRO-SER |
| 308 | Frequency of Salt-bridge formed between PRO-THR |
| 309 | Frequency of Salt-bridge formed between PRO-CYS |
| 310 | Frequency of Salt-bridge formed between PRO-MET |
| 311 | Frequency of Salt-bridge formed between PRO-ASN |
| 312 | Frequency of Salt-bridge formed between PRO-GLN |
| 313 | Frequency of Salt-bridge formed between PRO-ASP |
| 314 | Frequency of Salt-bridge formed between PRO-GLU |
| 315 | Frequency of Salt-bridge formed between PRO-LYS |
| 316 | Frequency of Salt-bridge formed between PRO-ARG |
| 317 | Frequency of Salt-bridge formed between PRO-HIS |
| 318 | Frequency of Salt-bridge formed between PHE-PHE |
| 319 | Frequency of Salt-bridge formed between PHE-TYR |
| 320 | Frequency of Salt-bridge formed between PHE-TRP |
| 321 | Frequency of Salt-bridge formed between PHE-SER |
| 322 | Frequency of Salt-bridge formed between PHE-THR |
| 323 | Frequency of Salt-bridge formed between PHE-CYS |
| 324 | Frequency of Salt-bridge formed between PHE-MET |
| 325 | Frequency of Salt-bridge formed between PHE-ASN |
| 326 | Frequency of Salt-bridge formed between PHE-GLN |
| 327 | Frequency of Salt-bridge formed between PHE-ASP |
| 328 | Frequency of Salt-bridge formed between PHE-GLU |
| 329 | Frequency of Salt-bridge formed between PHE-LYS |
| 330 | Frequency of Salt-bridge formed between PHE-ARG |
| 331 | Frequency of Salt-bridge formed between PHE-HIS |
| 332 | Frequency of Salt-bridge formed between TYR-TYR |
| 333 | Frequency of Salt-bridge formed between TYR-TRP |
| 334 | Frequency of Salt-bridge formed between TYR-SER |
| 335 | Frequency of Salt-bridge formed between TYR-THR |
| 336 | Frequency of Salt-bridge formed between TYR-CYS |
| 337 | Frequency of Salt-bridge formed between TYR-MET |
| 338 | Frequency of Salt-bridge formed between TYR-ASN |
| 339 | Frequency of Salt-bridge formed between TYR-GLN |
| 340 | Frequency of Salt-bridge formed between TYR-ASP |
| 341 | Frequency of Salt-bridge formed between TYR-GLU |
| 342 | Frequency of Salt-bridge formed between TYR-LYS |
| 343 | Frequency of Salt-bridge formed between TYR-ARG |
| 344 | Frequency of Salt-bridge formed between TYR-HIS |
| 345 | Frequency of Salt-bridge formed between TRP-TRP |
| 346 | Frequency of Salt-bridge formed between TRP-SER |
| 347 | Frequency of Salt-bridge formed between TRP-THR |
| 348 | Frequency of Salt-bridge formed between TRP-CYS |
| 349 | Frequency of Salt-bridge formed between TRP-MET |
| 350 | Frequency of Salt-bridge formed between TRP-ASN |
| 351 | Frequency of Salt-bridge formed between TRP-GLN |
| 352 | Frequency of Salt-bridge formed between TRP-ASP |
| 353 | Frequency of Salt-bridge formed between TRP-GLU |
| 354 | Frequency of Salt-bridge formed between TRP-LYS |
| 355 | Frequency of Salt-bridge formed between TRP-ARG |
| 356 | Frequency of Salt-bridge formed between TRP-HIS |
| 357 | Frequency of Salt-bridge formed between SER-SER |
| 358 | Frequency of Salt-bridge formed between SER-THR |
| 359 | Frequency of Salt-bridge formed between SER-CYS |
| 360 | Frequency of Salt-bridge formed between SER-MET |
| 361 | Frequency of Salt-bridge formed between SER-ASN |
| 362 | Frequency of Salt-bridge formed between SER-GLN |
| 363 | Frequency of Salt-bridge formed between SER-ASP |
| 364 | Frequency of Salt-bridge formed between SER-GLU |
| 365 | Frequency of Salt-bridge formed between SER-LYS |
| 366 | Frequency of Salt-bridge formed between SER-ARG |
| 367 | Frequency of Salt-bridge formed between SER-HIS |
| 368 | Frequency of Salt-bridge formed between THR-THR |
| 369 | Frequency of Salt-bridge formed between THR-CYS |
| 370 | Frequency of Salt-bridge formed between THR-MET |
| 371 | Frequency of Salt-bridge formed between THR-ASN |
| 372 | Frequency of Salt-bridge formed between THR-GLN |
| 373 | Frequency of Salt-bridge formed between THR-ASP |
| 374 | Frequency of Salt-bridge formed between THR-GLU |
| 375 | Frequency of Salt-bridge formed between THR-LYS |
| 376 | Frequency of Salt-bridge formed between THR-ARG |
| 377 | Frequency of Salt-bridge formed between THR-HIS |
| 378 | Frequency of Salt-bridge formed between CYS-CYS |
| 379 | Frequency of Salt-bridge formed between CYS-MET |
| 380 | Frequency of Salt-bridge formed between CYS-ASN |
| 381 | Frequency of Salt-bridge formed between CYS-GLN |
| 382 | Frequency of Salt-bridge formed between CYS-ASP |
| 383 | Frequency of Salt-bridge formed between CYS-GLU |
| 384 | Frequency of Salt-bridge formed between CYS-LYS |
| 385 | Frequency of Salt-bridge formed between CYS-ARG |
| 386 | Frequency of Salt-bridge formed between CYS-HIS |
| 387 | Frequency of Salt-bridge formed between MET-MET |
| 388 | Frequency of Salt-bridge formed between MET-ASN |
| 389 | Frequency of Salt-bridge formed between MET-GLN |
| 390 | Frequency of Salt-bridge formed between MET-ASP |
| 391 | Frequency of Salt-bridge formed between MET-GLU |
| 392 | Frequency of Salt-bridge formed between MET-LYS |
| 393 | Frequency of Salt-bridge formed between MET-ARG |
| 394 | Frequency of Salt-bridge formed between MET-HIS |
| 395 | Frequency of Salt-bridge formed between ASN-ASN |
| 396 | Frequency of Salt-bridge formed between ASN-GLN |
| 397 | Frequency of Salt-bridge formed between ASN-ASP |
| 398 | Frequency of Salt-bridge formed between ASN-GLU |
| 399 | Frequency of Salt-bridge formed between ASN-LYS |
| 400 | Frequency of Salt-bridge formed between ASN-ARG |
| 401 | Frequency of Salt-bridge formed between ASN-HIS |
| 402 | Frequency of Salt-bridge formed between GLN-GLN |
| 403 | Frequency of Salt-bridge formed between GLN-ASP |
| 404 | Frequency of Salt-bridge formed between GLN-GLU |
| 405 | Frequency of Salt-bridge formed between GLN-LYS |
| 406 | Frequency of Salt-bridge formed between GLN-ARG |
| 407 | Frequency of Salt-bridge formed between GLN-HIS |
| 408 | Frequency of Salt-bridge formed between ASP-ASP |
| 409 | Frequency of Salt-bridge formed between ASP-GLU |
| 410 | Frequency of Salt-bridge formed between ASP-LYS |
| 411 | Frequency of Salt-bridge formed between ASP-ARG* |
| 412 | Frequency of Salt-bridge formed between ASP-HIS |
| 413 | Frequency of Salt-bridge formed between GLU-GLU |
| 414 | Frequency of Salt-bridge formed between GLU-LYS |
| 415 | Frequency of Salt-bridge formed between GLU-ARG* |
| 416 | Frequency of Salt-bridge formed between GLU-HIS |
| 417 | Frequency of Salt-bridge formed between LYS-LYS |
| 418 | Frequency of Salt-bridge formed between LYS-ARG |
| 419 | Frequency of Salt-bridge formed between LYS-HIS |
| 420 | Frequency of Salt-bridge formed between ARG-ARG |
| 421 | Frequency of Salt-bridge formed between ARG-HIS |
| 422 | Frequency of Salt-bridge formed between HIS-HIS |
| 423 | Average Distance of Salt-bridge |
| 424 | Accessible surface area of chain 1 formed by LEU* |
| 425 | Accessible surface area of chain 1 formed by VAL* |
| 426 | Accessible surface area of chain 1 formed by ILE* |
| 427 | Accessible surface area of chain 1 formed by ALA* |
| 428 | Accessible surface area of chain 1 formed by GLY* |
| 429 | Accessible surface area of chain 1 formed by PRO* |
| 430 | Accessible surface area of chain 1 formed by PHE* |
| 431 | Accessible surface area of chain 1 formed by TYR* |
| 432 | Accessible surface area of chain 1 formed by TRP* |
| 433 | Accessible surface area of chain 1 formed by SER* |
| 434 | Accessible surface area of chain 1 formed by THR* |
| 435 | Accessible surface area of chain 1 formed by CYS* |
| 436 | Accessible surface area of chain 1 formed by MET* |
| 437 | Accessible surface area of chain 1 formed by ASN* |
| 438 | Accessible surface area of chain 1 formed by GLN* |
| 439 | Accessible surface area of chain 1 formed by ASP* |
| 440 | Accessible surface area of chain 1 formed by GLU* |
| 441 | Accessible surface area of chain 1 formed by LYS* |
| 442 | Accessible surface area of chain 1 formed by ARG* |
| 443 | Accessible surface area of chain 1 formed by HIS* |
| 444 | Accessible surface area of chain 2 formed by LEU* |
| 445 | Accessible surface area of chain 2 formed by VAL* |
| 446 | Accessible surface area of chain 2 formed by ILE* |
| 447 | Accessible surface area of chain 2 formed by ALA* |
| 448 | Accessible surface area of chain 2 formed by GLY* |
| 449 | Accessible surface area of chain 2 formed by PRO* |
| 450 | Accessible surface area of chain 2 formed by PHE* |
| 451 | Accessible surface area of chain 2 formed by TYR* |
| 452 | Accessible surface area of chain 2 formed by TRP* |
| 453 | Accessible surface area of chain 2 formed by SER* |
| 454 | Accessible surface area of chain 2 formed by THR* |
| 455 | Accessible surface area of chain 2 formed by CYS* |
| 456 | Accessible surface area of chain 2 formed by MET* |
| 457 | Accessible surface area of chain 2 formed by ASN* |
| 458 | Accessible surface area of chain 2 formed by GLN* |
| 459 | Accessible surface area of chain 2 formed by ASP* |
| 460 | Accessible surface area of chain 2 formed by GLU* |
| 461 | Accessible surface area of chain 2 formed by LYS* |
| 462 | Accessible surface area of chain 2 formed by ARG* |
| 463 | Accessible surface area of chain 2 formed by HIS* |
| 464 | Buried surface area of chain 1 formed by LEU |
| 465 | Buried surface area of chain 1 formed by VAL |
| 466 | Buried surface area of chain 1 formed by ILE* |
| 467 | Buried surface area of chain 1 formed by ALA |
| 468 | Buried surface area of chain 1 formed by GLY |
| 469 | Buried surface area of chain 1 formed by PRO |
| 470 | Buried surface area of chain 1 formed by PHE* |
| 471 | Buried surface area of chain 1 formed by TYR* |
| 472 | Buried surface area of chain 1 formed by TRP |
| 473 | Buried surface area of chain 1 formed by SER |
| 474 | Buried surface area of chain 1 formed by THR |
| 475 | Buried surface area of chain 1 formed by CYS |
| 476 | Buried surface area of chain 1 formed by MET |
| 477 | Buried surface area of chain 1 formed by ASN |
| 478 | Buried surface area of chain 1 formed by GLN |
| 479 | Buried surface area of chain 1 formed by ASP |
| 480 | Buried surface area of chain 1 formed by GLU* |
| 481 | Buried surface area of chain 1 formed by LYS |
| 482 | Buried surface area of chain 1 formed by ARG |
| 483 | Buried surface area of chain 1 formed by HIS |
| 484 | Buried surface area of chain 2 formed by LEU |
| 485 | Buried surface area of chain 2 formed by VAL |
| 486 | Buried surface area of chain 2 formed by ILE |
| 487 | Buried surface area of chain 2 formed by ALA |
| 488 | Buried surface area of chain 2 formed by GLY |
| 489 | Buried surface area of chain 2 formed by PRO |
| 490 | Buried surface area of chain 2 formed by PHE |
| 491 | Buried surface area of chain 2 formed by TYR |
| 492 | Buried surface area of chain 2 formed by TRP |
| 493 | Buried surface area of chain 2 formed by SER |
| 494 | Buried surface area of chain 2 formed by THR |
| 495 | Buried surface area of chain 2 formed by CYS |
| 496 | Buried surface area of chain 2 formed by MET |
| 497 | Buried surface area of chain 2 formed by ASN |
| 498 | Buried surface area of chain 2 formed by GLN |
| 499 | Buried surface area of chain 2 formed by ASP |
| 500 | Buried surface area of chain 2 formed by GLU |
| 501 | Buried surface area of chain 2 formed by LYS* |
| 502 | Buried surface area of chain 2 formed by ARG |
| 503 | Buried surface area of chain 2 formed by HIS |
| 504 | Total Buried surface area formed by LEU |
| 505 | Total Buried surface area formed by VAL |
| 506 | Total Buried surface area formed by ILE* |
| 507 | Total Buried surface area formed by ALA |
| 508 | Total Buried surface area formed by GLY |
| 509 | Total Buried surface area formed by PRO |
| 510 | Total Buried surface area formed by PHE* |
| 511 | Total Buried surface area formed by TYR* |
| 512 | Total Buried surface area formed by TRP* |
| 513 | Total Buried surface area formed by SER |
| 514 | Total Buried surface area formed by THR |
| 515 | Total Buried surface area formed by CYS |
| 516 | Total Buried surface area formed by MET |
| 517 | Total Buried surface area formed by ASN |
| 518 | Total Buried surface area formed by GLN |
| 519 | Total Buried surface area formed by ASP |
| 520 | Total Buried surface area formed by GLU* |
| 521 | Total Buried surface area formed by LYS |
| 522 | Total Buried surface area formed by ARG |
| 523 | Total Buried surface area formed by HIS |

Common distinguishable features are marked by *

**Table S3: Parameters of kernel function in different training models**

| Model | SVM Type | Kernel Type | C value  (Error control factor) | gamma (Decision boundary factor) | No. of Class | Total support vectors | rho(Bias weight,  *-b*) | No. of vector falling on boundary of class 1 | No. of vector falling on boundary of class 2 |
| --- | --- | --- | --- | --- | --- | --- | --- | --- | --- |
| hetero_FNAT_moderately_train.scale.model | c_svc | RBF | 8 | 0.03125 | 2 | 411 | -0.220798 | 161 | 161 |
| hetero_FNAT_mixed_train.scale.model | c_svc | RBF | 8192 | 0.0078125 | 2 | 317 | 0.414445 | 147 | 147 |
| hetero_FNAT_highly_train.scale.model | c_svc | RBF | 8192 | 0.03125 | 2 | 430 | -0.223802 | 192 | 192 |
| hetero_FNAT_weakly_train.scale.model | c_svc | RBF | 8 | 0.03125 | 2 | 459 | -0.260012 | 169 | 169 |
| hetero_iRMSD_moderately_train.scale.model | c_svc | RBF | 32 | 0.0078125 | 2 | 393 | -3.21157 | 174 | 174 |
| hetero_iRMSD_mixed_train.scale.model | c_svc | RBF | 128 | 0.0001221 | 2 | 254 | 10.3111 | 128 | 128 |
| hetero_iRMSD_highly_train.scale.model | c_svc | RBF | 32 | 0.0078125 | 2 | 314 | -0.158682 | 136 | 136 |
| hetero_iRMSD_weakly_train.scale.model | c_svc | RBF | 32768 | 3.052E-05 | 2 | 312 | 78.7934 | 160 | 160 |
| homo_FNAT_moderately_train.scale.model | c_svc | RBF | 8 | 0.03125 | 2 | 378 | -1.08357 | 147 | 147 |
| homo_FNAT_mixed_train.scale.model | c_svc | RBF | 32 | 0.0078125 | 2 | 337 | -4.98258 | 150 | 150 |
| homo_FNAT_highly_train.scale.model | c_svc | RBF | 8 | 0.03125 | 2 | 301 | -0.989011 | 112 | 112 |
| homo_FNAT_weakly_train.scale.model | c_svc | RBF | 32 | 0.0078125 | 2 | 402 | -7.38716 | 171 | 171 |
| homo_iRMSD_moderately_train.scale.model | c_svc | RBF | 8192 | 0.03125 | 2 | 307 | -0.447667 | 126 | 126 |
| homo_iRMSD_mixed_train.scale.model | c_svc | RBF | 8192 | 0.03125 | 2 | 462 | -0.727689 | 191 | 191 |
| homo_iRMSD_highly_train.scale.model | c_svc | RBF | 8192 | 0.03125 | 2 | 244 | -0.287839 | 91 | 91 |
| homo_iRMSD_weakly_train.scale.model | c_svc | RBF | 128 | 0.0019531 | 2 | 283 | -4.64627 | 130 | 130 |
